# The TRPA1 Agonist Cinnamaldehyde Induces the Secretion of HCO_3_^−^ by the Porcine Colon

**DOI:** 10.3390/ijms22105198

**Published:** 2021-05-14

**Authors:** David Manneck, Gisela Manz, Hannah-Sophie Braun, Julia Rosendahl, Friederike Stumpff

**Affiliations:** 1 Department of Veterinary Medicine, Institute of Veterinary Physiology, Freie Universität Berlin, Oertzenweg 19b, 14163 Berlin, Germany; david.manneck@fu-berlin.de (D.M.); gisela.manz@fu-berlin.de (G.M.); 2 PerformaNat GmbH, Hohentwielsteig 6, 14163 Berlin, Germany; hannah-sophie.braun@fu-berlin.de (H.-S.B.); rosendahl@performanat.de (J.R.)

**Keywords:** cinnamaldehyde, colon, colonic buffering, epithelial transport, essential oils, intestine, patch clamp, pig, prostaglandin, TRPA1, TRPV3, Ussing chamber

## Abstract

A therapeutic potential of the TRPA1 channel agonist cinnamaldehyde for use in inflammatory bowel disease is emerging, but the mechanisms are unclear. Semi-quantitative qPCR of various parts of the porcine gastrointestinal tract showed that mRNA for TRPA1 was highest in the colonic mucosa. In Ussing chambers, 1 mmol·L^−1^ cinnamaldehyde induced increases in short circuit current (ΔI_sc_) and conductance (ΔG_t_) across the colon that were higher than those across the jejunum or after 1 mmol·L^−1^ thymol. Lidocaine, amiloride or bumetanide did not change the response. The application of 1 mmol·L^−1^ quinidine or the bilateral replacement of 120 Na^+^, 120 Cl^−^ or 25 HCO_3_^−^ reduced ΔG_t_, while the removal of Ca^2+^ enhanced ΔG_t_ with ΔI_sc_ numerically higher. ΔI_sc_ decreased after 0.5 NPPB, 0.01 indometacin and the bilateral replacement of 120 Na^+^ or 25 HCO_3_^−^. The removal of 120 Cl^−^ had no effect. Cinnamaldehyde also activates TRPV3, but comparative measurements involving patch clamp experiments on overexpressing cells demonstrated that much higher concentrations are required. We suggest that cinnamaldehyde stimulates the secretion of HCO_3_^−^ via apical CFTR and basolateral Na^+^-HCO_3_^−^ cotransport, preventing acidosis and damage to the epithelium and the colonic microbiome. Signaling may involve the opening of TRPA1, depolarization of the epithelium and a rise in PGE2 following a lower uptake of prostaglandins via OATP2A1.

## 1. Introduction

The transient receptor potential ankyrin channel (TRPA1) is a non-selective member of the large family of transient receptor potential (TRP) channels that is expressed by sensory neurons, epithelia and a wide variety of other cells, where it plays a key role as a sensor of multiple external and internal stimuli. In comparison to most other members of the TRP channel family, TRPA1 has a fairly high permeability to Ca^2+^, with P(Na^+^)/P(Ca^2+^) ~ 6, a value that can rise to about nine when the channel is opened by an agonist [1]. The selectivity for monovalent cations follows an Eisenman XI sequence with Na^+^ > K^+^ [2,3], with permeation dropping when Ca^2+^ or other divalent cations are present [4].

The name of the channel reflects the presence of 14–18 ankyrin repeats at its very long cytosolic NH_2_ terminus, a distinct feature that is thought to be relevant for its promiscuous interaction with a very large number of dramatically different stimuli [1,2]. TRPA1 opens not only in response to intensive cold, but also to pungent compounds contained in certain plants, such as allyl isothiocyanate (AITC, contained in mustard oil), cinnamaldehyde and thymol. Furthermore, TRPA1 is a sensor for hyper- and hypoxia, various reactive oxygen species (ROS), H_2_S, certain prostaglandins and an immense number of other chemical species and endogenous signals, many of which are associated with cell damage, and are released in acute and chronic pain and inflammation. Accordingly, TRPA1 is involved in the pathophysiology of multiple organs [1,2].

A dominant role in the sensation pain is confirmed by the major symptom in the only TRPA1 channelopathy known at this point [5]. In what is known as Familial Episodic Pain Syndrome (FEPS), a gain of function mutation of TRPA1 causes episodes of severe pain localized principally to the upper body that are triggered by cold, fasting or physical stress. Interestingly, the baseline pain thresholds are not impaired. Furthermore, it has emerged that hyperalgesia can involve the direct action of mediators of oxidative stress on TRPA1 channels in addition to the classical receptor-mediated cascades [6,7,8].

The debate is ongoing concerning the function of TRPA1 in the intestinal tract. TRPA1 is expressed by sensory extrinsic and intrinsic afferent neurons that innervate the viscera [9,10,11], by intestinal myenteric and motor neurons which control motility [1,12,13], and by endocrine and transporting cells of the epithelial mucosa [14,15,16]. Although visceral symptoms have not been reported in gain of function mutations of TRPA1 [5], there is a clear association with visceral hypersensitivity which can be purely functional or associated with diseases such as colitis ulcerosa or Crohn’s disease [10,17]. Apart from direct activation by prostaglandin and its metabolites [6,7,8], TRPA1 can also be activated by immunostimulatory cues such as the lipopolysaccharides (LPS) or outer wall glycolipids that are released by gram-negative bacteria after lysis [18]. Accordingly, in mouse models, tail-flick hyperalgesia and a fall in blood pressure is observed within a minute of LPS injection, which is clearly the result of the activation of neuronal afferents long before the production of immunomodulators such as TNF-α sets in [9,18].

Somewhat curiously and in contrast to their aversive role in signalling cellular damage in pain and inflammation, TRPA1 agonists in the form of spices have played an important role in the culinary arts for millenia [1]. Furthermore, grazers are known to show a preference for certain herbal compounds which activate TRPA1 and other TRP channels, but the reasons for this are unclear [19]. Having evolved to protect plants from bacteria, fungi and viruses, TRP channel modulators clearly have a multi-target antimicrobial potential [20]. However, the amounts that are voluntarily consumed by humans and animals are far lower than those required to achieve significant antibacterial effects.

In humans, the activation of TRPA1 has been suggested to lead to feelings of satiety after the ingestion of fragrant compounds found in spices such as cinnamaldehyde [21,22], allicin [23], menthol [24], or thymol [25], all of which directly activate the channel in vitro. Furthermore, these TRPA1 agonists were found to facilitate the secretion of cholecystokinin (CCK) and 5-HT from enteroendocrine cells (EEC), and thus to enhance the digestive response [26,27]. In addition, a growing body of literature suggests that TRPA1 agonists have anti-inflammatory effects with therapeutic potential in bowel diseases [20,28]. Thus, cinnamaldehyde was found to attenuate experimental colitis induced by 2,4,6-trinitrobenzene sulfonic or acetic acid in rat models of the human disease, with the reduction both of the symptoms and various markers of inflammation, such as TNF-α, myeloperoxidase and IL-6 [29,30]. While various signalling cascades are being discussed, it is striking that cinnamaldehyde activates TRPA1 with a specificity that surpasses that of the classical TRPA1 agonist AITC [1].

In farm animals, modulators of TRP channels in general and of TRPA1 in particular are increasingly being added to the feed of livestock as an alternative to antibiotic growth enhancers [31,32]. This is particularly important during weaning, during which young animals that have previously obtained readily digestible milk from their mothers are switched to diets rich in plant fiber. Because the structural carbohydrates contained in plants cannot be broken down by the mammalian enzymes found in the small intestine, after weaning, large quantities of undigested material suddenly begin to enter the caecum and the colon. Here, a growing community of bacteria and fungi break up previously undigested material, producing energy-rich short chain fatty acids that can then be absorbed and utilized by the young animal to produce glucose and other energy-rich carbohydrates within the liver [33,34]. However, in the process, large quantities of protons are set free and have to be buffered in the hindgut lumen or within the cytosol to prevent epithelial damage. While the bacterial colonization of the hindgut is thus central for the survival and growth of the young animal, the transition can be harsh and many piglets develop inflammatory responses with severe or even lethal diarrhea [35,36].

Given the importance of the pig both in food production and as a model species for research on humans, it was the purpose of this study to compare the expression of TRPA1 in the various segments of the gastrointestinal tract via qPCR, and to identify segments with high expression. In order to assess the functional importance of TRPA1 in the segment with the highest level of expression, Ussing chamber experiments were performed on colonic epithelia using cinnamaldehyde, a classical agonist of TRPA1 with possible therapeutic potential [1,2,29,30]. Using the whole cell configuration of the patch clamp technique on HEK-293 cells overexpressing TRPV3, we investigated a potential additional contribution of TRPV3 to the cinnamaldehyde response [37]. A more detailed understanding of the mechanisms of action of this phytogenic substance could be useful for a better understanding of colonic function, but also for finding new therapy options for the rising number of individuals suffering from inflammatory bowel disease worldwide [38].

## 2. Results

### 2.1. PCR

**Figure 1:**
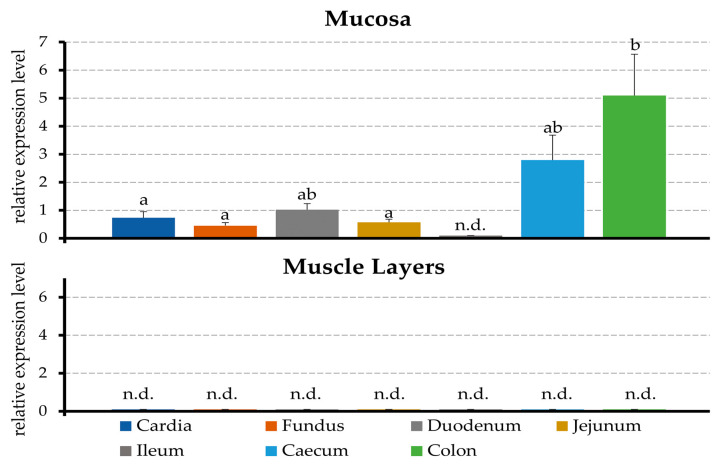
Relative mRNA expression of TRPA1 in the mucosa of the stomach (fundus and cardia), duodenum, jejunum, ileum, caecum and colon, and associated muscle layers of four young pigs from a controlled in-house study. Normalization was performed to the reference genes ACTB, GAPDH and YWHAZ, with scaling to the mean values of all of the samples. The letters above the bars indicate statistically significant differences between those bars that do not share a letter (*p* < 0.05) (n.d. = not detected).

RT-qPCR was used to investigate the relative amounts of mRNA encoding for TRPA1 in the gastrointestinal tissues of stomach (fundus and cardia), duodenum, jejunum, ileum, caecum, and colon of four pigs. The expression was analyzed in the mucosa and tunica muscularis; normalized to the reference genes ACTB, GAPDH, and YWHAZ; and scaled to the mean value of all of the samples. Messenger RNA encoding for TRPA1 could be detected in all of the gastrointestinal epithelia investigated except for the ileum. The expression of TRPA1 by the colonic epithelium varied strongly depending on the animal, and was significantly higher than that of all other epithelia, except for the duodenum and caecum. In contrast, any expression of TRPA1 by the muscular layers was under the limit of detection (Figure 1).

### 2.2. Ussing Chamber Studies

#### 2.2.1. Effect of Cinnamaldehyde

**Figure 2:**
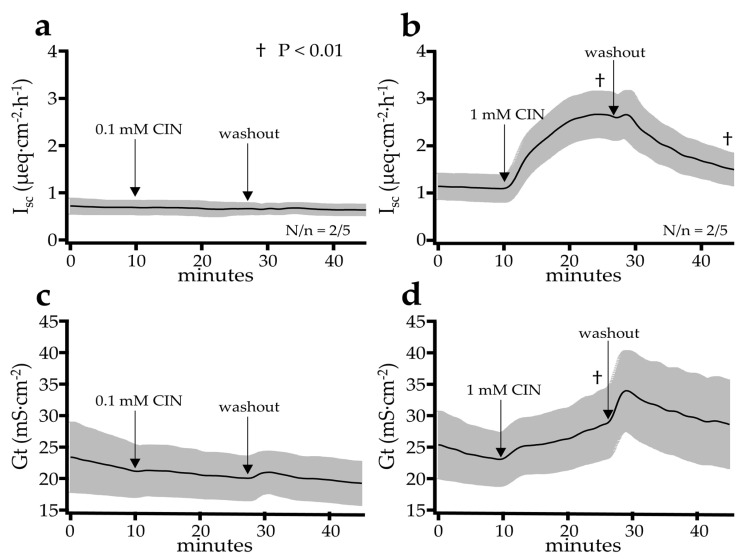
Effect of the TRPA1 agonist cinnamaldehyde at a concentration of 100 µmol·L^−1^ (**a**,**c**) and 1 mmol·L^−1^ (**b**,**d**) on I_sc_ and G_t_ in the Ussing chamber using the colonic tissue of young pigs (controlled in-house study). While the smaller concentration of 100 µmol·L^−1^ was insufficient, after the addition of 1 mmol·L^−1^, a significant increase of the I_sc_ and G_t_ could be observed. After washout, the values dropped again. N/n = the number of animals/number of tissues, which was identical for G_t_ and I_sc_.

Cinnamaldehyde is known as a classical agonist of TRPA1 with high specificity [1,2]. The functional expression of TRPA1 was tested via the response to the bilateral application of 100 µmol·L^−1^ or 1 mmol·L^−1^ cinnamaldehyde in the colon of two pigs with a total of five tissues in a first set of experiments. After an incubation period of 15 min, washout was performed. The addition of 100 µmol·L^−1^ cinnamaldehyde showed no effect on the short circuit current I_sc_ and tissue conductance G_t_ (*p* > 0.1) (Figure 2). Before the addition of cinnamaldehyde, the I_sc_ remained relatively constant with 0.69 ± 0.15 µeq·cm^−2^·h^−1^ immediately before addition, reaching a peak value of 0.66 ± 0.13 µeq·cm^−2^·h^−1^ within the 15 min period after the addition (*p* > 0.1). The conductance also essentially remained constant, with a value of 21.2 ± 4.14 mS·cm^−2^ before and 20.2 ± 3.57 mS·cm^−2^ 15 min after the addition of cinnamaldehyde (*p* > 0.1). Visible and statistically significant effects on I_sc_ and G_t_ were observed after the addition of cinnamaldehyde in a higher concentration. Within the 15 min period after the application of cinnamaldehyde in a concentration of 1 mmol·L^−^^1^, the I_sc_ increased from 1.10 ± 0.29 µeq·cm^−2^·h^−1^ to a peak value of 2.66 ± 0.49 µeq·cm^−2^·h^−1^, after which, in some tissues, a decline could be observed. The conductance rose continuously in all of the tissues from 23.1 ± 4.20 mS·cm^−2^ to 28.4 ± 5.42 mS·cm^−2^ at the end of the 15 min period (both *p* < 0.05). After the washout, I_sc_ returned to 1.31 ± 0.33 µeq·cm^−2^·h^−1^ and the conductance returned to 26.9 ± 6.60 mS·cm^−2^, values that were not significantly different from the baseline at the start of the experiment (*p* > 0.1). We also performed similar experiments in the jejunum, in which 1 mmol·L^−1^ cinnamaldehyde again led to significant rises in I_sc_ (ΔI_sc_, *p* = 0.029) and a trend for a rise in G_t_ (∆G_t_, *p* = 0.079). Interestingly, the effects tended to be smaller than those observed in the colon (*p* = 0.015 for ΔI_sc_ and *p* = 0.063 for ∆G_t_) (see Supplementary Materials Figure S1).

**Figure 3:**
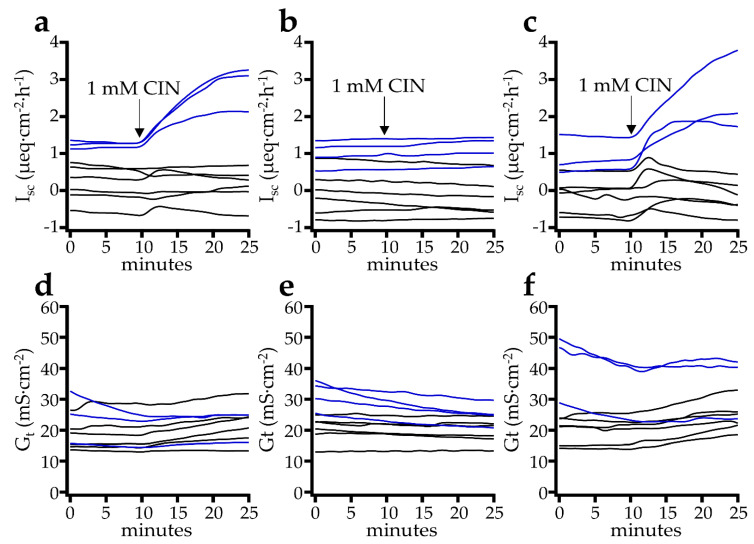
Effect of the TRPA1 agonist cinnamaldehyde (CIN) at a concentration of 1 mmol·L^−1^ after the mucosal (**a**,**d**), serosal (**b**,**e**) or bilateral addition (**c**,**f**) to I_sc_ and G_t_ in the Ussing chamber using the colonic tissue of pigs. After mucosal and bilateral addition, a significant increase in I_sc_ and G_t_ was observed, although the effects were absent after the serosal addition (details see text). In this figure, some tissues were obtained from older, larger pigs from a commercial slaughterhouse (blue lines), which showed a strong, sustained increase, while the black lines reflect the frequently biphasic responses that were seen in younger and smaller pigs slaughtered within a controlled study, as in the rest of the manuscript.

In the next set of screening experiments, we examined the response to 1 mmol·L^−1^ cinnamaldehyde after the mucosal, serosal, or bilateral addition in the colon of five pigs with a total of nine or ten tissues per group (Figure 3). As in the previous series of experiments, we observed a rapid increase in I_sc_ from 0.59 ± 0.50 µeq·cm^−2^·h^−1^ to peak values of 1.39 ± 0.71 µeq·cm^−2^·h^−1^ (*p* = 0.016) within a 15 min period after the bilateral application of cinnamaldehyde, with a slightly slower increase in G_t_ from 24.8 ± 3.21 mS·cm^−2^ to 28.0 ± 2.83 mS·cm^−^^2^ (*p* = 0.006) at the end of the 15 min period. The same effect was also observed after the mucosal application, in which I_sc_ increased from 0.47 ± 0.23 µeq·cm^−2^·h^−1^ to 1.09 ± 0.46 µeq·cm^−^^2^·h^−^^1^ (*p* = 0.012) and G_t_ from 19.2 ± 1.81 mS·cm^−2^ to 21.8 ± 1.87 mS·cm^−2^ (*p* = 0.007). No effect on I_sc_ was observed after serosal application, which changed only slightly from 0.34 ± 0.24 µeq·cm^−2^·h^−1^ to 0.34 ± 0.25 µeq·cm^−2^·h^−1^ in the 15 min period (*p* = 0.85). G_t_ dropped from 23.2 ± 1.80 mS·cm^−2^ to 21.5 ± 1.46 mS·cm^−2^ during the same period (*p* = 0.013). However, the slope of the curve did not change after the application of cinnamaldehyde, so that this most likely reflects a baseline drift. Again, the response in the jejunum was different from that in the colon, where we observed increases in I_sc_ not only after mucosal and bilateral addition, but also after the serosal addition. Conversely, any effects of cinnamaldehyde on the G_t_ of the jejunum were subtle (see Supplementary Materials Figure S2).

Finally, it should be mentioned that the tissues from three animals from a commercial slaughterhouse showed no response to cinnamaldehyde despite a positive reaction to theophylline (data not included). In these cases, there was a delay in the removal of the tissue from the carcass. It appears possible that cells from the absorptive surface epithelium (most exposed to gastrointestinal toxins and the major locus of TRPA1 expression [39]) were damaged more severely than the cells found within the crypts, where theophylline-induced secretion occurs. The subsequent experiments were therefore performed with tissues rapidly removed from piglets euthanized within a controlled study.

#### 2.2.2. Effect of Blockers on the Cinnamaldehyde Response

**Figure 4:**
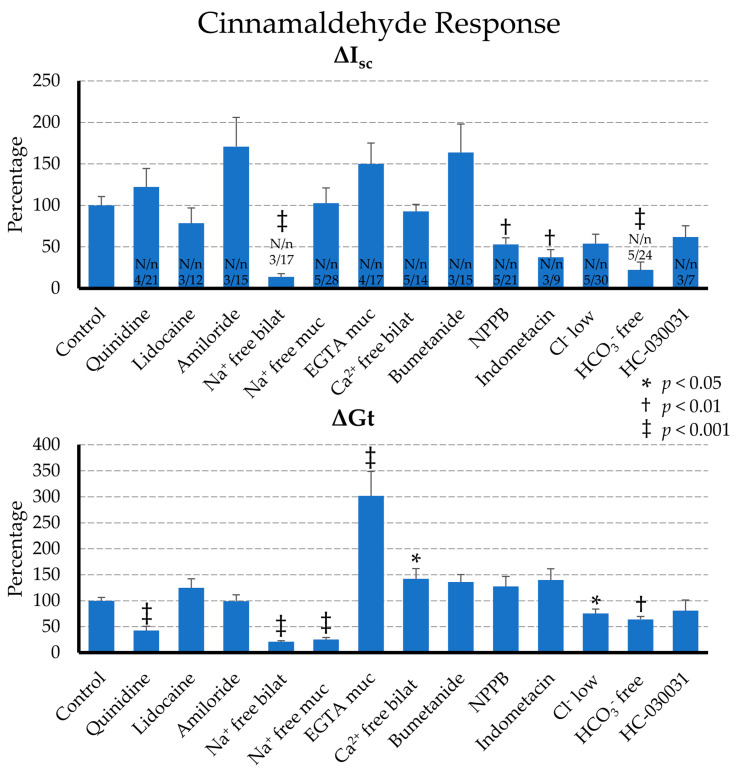
Comparison of the effect of cinnamaldehyde (1 mmol·L^−1^) on the short circuit current (ΔI_sc_) and the conductance (ΔG_t_) of the colonic mucosa from young pigs (controlled in-house study) in Ussing chambers after preincubation with different blockers, or after the ion replacement. In order to allow a comparison of the data from different sets of experiments, the differences (deltas) were calculated by subtracting the peak value of the cinnamaldehyde response in the 15 min period after addition of cinnamaldehyde from the baseline value before addition. The delta values of the control tissues of each animal were set to 100%, and the deltas of the treated tissues were calculated as a percentage of the control tissues of the animal in question. Significant differences versus the control group are marked as *, †, or ‡ (*p* < 0.05, *p* < 0.01 or *p* < 0.001). For the concentrations of the blockers and the composition of the solutions used, see the results and the Supplementary Materials. N/n = number of animals/number of tissues, which were identical for G_t_ and I_sc_; muc = mucosal; bilat = bilateral.

In order to study the effect of cinnamaldehyde in a little more detail, we treated colonic tissues with different blockers 15 min before the addition of cinnamaldehyde to assess the response after the corresponding pretreatment. For each treatment, control tissues from the same animals were given the corresponding amount of solvent and served as a comparison. Delivery to the epithelium even in situations with the ample formation of mucus was ensured via bilateral application of cinnamaldehyde. The baseline parameters before and after the pretreatment are given in Table 1, while the data from before and after the cinnamaldehyde application are given in Table 2. The control values in the tables are the means of all of the control tissues used. The difference in the values before and after cinnamaldehyde application (ΔI_sc_ and ΔG_t_) are given in the text, along with the respective control values. Because the animals in the different data sets showed individual variability, ΔI_sc_ and ΔG_t_ are also given as a percentage of the control value for each data set from the same group of animals in Figure 4.

Neuronal involvement was tested in a first set of tissues (N/n = 3/12; number of animals/number of tissues), which were preincubated with serosal lidocaine (1 mmol·L^−1^) before the bilateral addition of cinnamaldehyde (1 mmol^−1^). The addition of lidocaine visibly decreased the baseline I_sc_ by ΔI_sc_ = −0.14 ± 0.021 µeq·cm^−2^·h^−1^ (*p* < 0.001), with little change in G_t_ (which numerically shifted by ΔG_t_ = −0.87 ± 0.50 mS·cm^−2^; *p* = 0.15) (Table 1). No response was seen in the control group (N/n = 3/12). After the addition of cinnamaldehyde, an identical increase in I_sc_ and G_t_ could be observed in both groups (*p* > 0.1). In the lidocaine group, I_sc_ increased by ΔI_sc_ = 0.78 ± 0.18 µeq·cm^−2^·h^−1^ (or 78.6 ± 18.3% of the response of the control group, which was set to 100%), while the change in conductance (ΔG_t_) was 6.41 ± 0.87 mS·cm^−2^ (or 125.0 ± 17.0% of the control; N/n = 3/12).

In a second set of tissues, preincubation with 1 mmol·L^−1^ mucosal quinidine was used (N/n = 4/21) as a blocker of non-selective cation channels. The addition of quinidine resulted in a significant decrease in the baseline I_sc_ by ΔI_sc_ = −0.17 ± 0.022 µeq·cm^−2^·h^−1^, and of G_t_ by ΔG_t_ = −2.52 ± 0.35 mS·cm^−2^ (both *p* < 0.001). After the subsequent addition of cinnamaldehyde, a ΔI_sc_ response of 0.96 ± 0.17 µeq·cm^−2^·h^−1^ was observed, which remained at 122.4 ± 22.1% of the control (N/n = 4/23, *p* > 0.1). However, the ΔG_t_ response of 1.74 ± 0.34 mS·cm^−2^ (42.6 ± 8.3% of the control) was reduced strongly by more than half (*p* < 0.001).

Another set of epithelia was treated with 1 mmol·L^−1^ mucosal amiloride (N/n = 3/15), which decreased the baseline I_sc_ by ΔI_sc_ = −0.26 ± 0.055 µeq·cm^−2^·h^−1^ and G_t_ by ΔG_t_ = −1.77 ± 0.38 mS·cm^−2^ (both *p* < 0.05), most likely reflecting a block of the epithelial sodium channel ENaC (SCNN1). After cinnamaldehyde addition, ΔI_sc_ was 1.00 ± 0.21 µeq·cm^−2^·h^−1^ (170.8 ± 35.1% of the control, N/n = 3/8) and ΔG_t_ was 3.70 ± 0.45 mS·cm^−2^ (99.2 ± 12.2% of the control), both of which were not different from the control tissues (*p* > 0.1).

In order to selectively inhibit TRPA1, we used the antagonist HC-030031 with a concentration of 100 µmol·L^−1^ on both sides (N/n = 3/7). In rat colon, this concentration of HC-030031 blocked the cinnamaldehyde response [39]. Again, we observed a small but significant decrease in the baseline I_sc_ of ΔI_sc_ = −0.063 ± 0.022 µeq·cm^−2^·h^−1^ after the preincubation period (*p* = 0.028), with the baseline G_t_ remaining unchanged (*p* > 0.1). The application of cinnamaldehyde (1 mmol·L^−1^) resulted in a numerically smaller ΔI_sc_ of 0.63 ± 0.14 µeq·cm^−2^·h^−1^ (or 61.8 ± 13.5% of the control, N/n = 3/18), but the effects did not show significance (*p* = 0.138). The ΔG_t_ remained at 4.41 ± 1.10 mS·cm^−2^ (81.0 ± 20.1% of the control).

An increase in I_sc_ may reflect either cation absorption or anion secretion, with the latter being classically driven by the Na^+^-K^+^-2Cl^−^ cotransporter 1 (NKCC1) in the colon. Since NKCC1 is blocked by bumetanide, 1 mmol·L^−1^ were added to the serosal side (N/n = 3/15), which decreased the baseline I_sc_ by ΔI_sc_ = −0.10 ± 0.023 µeq·cm^−2^·h^−1^ (*p* < 0.001). The baseline G_t_ was unchanged (ΔG_t_ = 0.035 ± 0.23 mS·cm^−2^, *p* > 0.1). After the addition of cinnamaldehyde, I_sc_ increased by ΔI_sc_ = 0.96 ± 0.20 µeq·cm^−2^·h^−1^ and G_t_ by ΔG_t_ = 5.07 ± 0.53 mS·cm^−2^, values that were not different from the corresponding control (163.7 ± 34.4% and 135.9 ± 19.2%, respectively; *p* > 0.1, N/n = 3/8).

Further tissues were treated with the anion channel blocker NPPB at a concentration of 0.5 mmol·L^−1^ on the mucosal side (N/n = 5/21), which decreased the baseline I_sc_ by ΔI_sc_ = −0.20 ± 0.047 µeq·cm^−2^·h^−1^ (*p* < 0.001) and the baseline G_t_ by ΔG_t_ = −0.95 ± 0.32 mS·cm^−2^ (*p* < 0.008). After the subsequent addition of cinnamaldehyde, a significantly smaller ΔI_sc_ was observed: 0.45 ± 0.07 µeq·cm^−2^·h^−1^ (or 53.1 ± 7.87% of the control, *p* = 0.06). However, the ΔG_t_ response remained unchanged at 5.73 ± 0.86 mS·cm^−2^ (127.5 ± 19.2% of the control, N/n = 5/29).

In order to investigate whether the cinnamaldehyde response involves prostaglandin signalling, 10 µmol·L^−1^ indometacin was added to both sides to inhibit cyclooxygenases (N/n = 3/9). A slight decrease in the baseline I_sc_ level was observed by ΔI_sc_ = −0.079 ± 0.032 µeq·cm^−2^·h^−1^ (*p* = 0.04), with no effect on G_t_. In response to cinnamaldehyde, a significantly smaller ΔI_sc_ of only 0.39 ± 0.10 µeq·cm^−2^·h^−1^ or 37.5 ± 9.3% of the control was observed (*p* = 0.003, N/n = 3/18), while the ΔG_t_ of 7.63 ± 1.17 mS·cm^−2^ (140.1 ± 21.5% of the control) was not significantly changed by indometacin.

#### 2.2.3. Effect of Ion Replacement on the Cinnamaldehyde Response

In a second experimental section, we replaced certain ions in the solutions in order to evaluate the effect of cations and anions on the cinnamaldehyde response (see Suplementary Materials Table S1). In the control tissues, a sham solution change was performed. Again, all of the results are summarized in Table 1 and Table 2, and in Figure 4.

First, we switched to a Na^+^-free solution 15 min before the addition of cinnamaldehyde on both sides (N/n = 3/17), replacing sodium with equivalent amounts of NMDG^+^. This led to a sharp drop of I_sc_ by ΔI_sc_ = −0.81 ± 0.063 µeq·cm^−2^·h^−1^ (or from 0.42 ± 0.066 to −0.40 ± 0.052 µeq·cm^−2^·h^−1^), while the G_t_ dropped by about half (ΔG_t_ = −9.30 ± 1.17 mS·cm^−2^, from 18.2 ± 1.58 to 8.9 ± 0.53 mS·cm^−2^, both *p* < 0.001). However, even in the bilateral absence of Na^+^, an increase in I_sc_ and G_t_ could still be observed after the application of cinnamaldehyde of ΔI_sc_ = 0.14 ± 0.039 µeq·cm^−2^·h^−1^ (*p* = 0.002) and ΔG_t_ = 1.16 ± 0.10 mS·cm^−2^ (*p* < 0.001), respectively. However, the magnitude of the response to cinnamaldehyde was strongly reduced, with ΔI_sc_ at 13.8 ± 3.8% of the control (N/n = 3/18) and ΔG_t_ at only 21.2 ± 1.84% of the control (both *p* < 0.001).

The replacement of Na^+^ on the mucosal side only (N/n = 5/28) decreased the basal I_sc_ by ΔI_sc_ = −1.03 ± 0.076 µeq·cm^−2^·h^−1^ (or from 0.34 ± 0.044 to −0.69 ± 0.062 µeq·cm^−2^·h^−^^1^) and the basal G_t_ by ΔG_t_ = −5.41 ± 0.77 mS·cm^−2^ (or from 17.6 ± 0.95 to 12.2 ± 0.39 mS·cm^−2^) (both *p* < 0.001). After the addition of cinnamaldehyde, a G_t_ response occurred with a ΔG_t_ of 1.15 ± 0.17 mS·cm^−2^ (*p* < 0.001), which corresponded to 25.3 ± 3.67% of the control response (N/n = 5/29, *p* < 0.001). This response was identical to that observed after the bilateral removal of Na^+^ (*p* = 0.96). In marked contrast, ΔI_sc_ was not affected, but remained at 0.51 ± 0.089 µeq·cm^−^^2^·h^−^^1^, or 102.8 ± 18.2% of the control (*p* > 0.1).

In the next step, we wanted to investigate the involvement of the divalent cation Ca^2+^. For this purpose, a set of epithelia was changed mucosally to a calcium-free solution with EGTA (N/n = 4/17). Otherwise, the composition of the NaCl solution remained identical on both sides. The basal level I_sc_ increased by ΔI_sc_ = 0.18 ± 0.060 µeq·cm^−2^·h^−1^ (*p* = 0.01), reflecting the stimulation of a transcellular transport mechanism. The conductance G_t_ increased by ΔG_t_ = 4.70 ± 0.85 mS·cm^−2^ (*p* < 0.001). The subsequent application of cinnamaldehyde induced a ΔI_sc_ of 1.18 ± 0.20 µeq·cm^−2^·h^−1^ or 150.0 ± 25.1% of the control tissues from the pigs of the series (N/n = 4/17). When tested against all of the control tissues investigated (N/n = 12/70), this difference tested for significance (*p* = 0.022). The G_t_ response to cinnamaldehyde rose dramatically after the removal of calcium, with ΔG_t_ at 12.3 ± 1.92 mS·cm^−2^ or 301.8 ± 47.2% of the control (*p* < 0.001).

In a second similar set of experiments, we removed Ca^2+^ on both sides (N/n = 5/14). In order to prevent damage to the epithelium, the solution did not contain EGTA. No increase in the baseline I_sc_ was observed as a result of this pretreatment, although G_t_ increased by 1.05 ± 0.35 mS·cm^−2^ (*p* = 0.011). This suggests that here, too, the rate of transcellular transport must have increased to compensate for paracellular leak currents. The response to cinnamaldehyde did not change and ΔI_sc_ remained at 0.79 ± 0.072 µeq·cm^−2^·h^−1^ (or 92.8 ± 8.37% of the control), although ΔG_t_ increased by 6.40 ± 0.87 mS·cm^−2^ (142.3 ± 19.4% of the control, *p* = 0.042).

The involvement of anions in the cinnamaldehyde response was tested by incubating the epithelia in a solution with a low Cl^−^ concentration (10.3 instead of 130.3 mmol·L^−1^) or in a HCO_3_^−^-free solution (buffered only with HEPES). Because these epithelia were incubated with the test solution from the beginning of the experiment, the baseline I_sc_ and G_t_ levels were compared to the control tissues.

Interestingly, the tissues in the low Cl^−^ solution (N/n = 5/30) had a higher mean baseline I_sc_ level of 0.52 ± 0.071 µeq·cm^−2^·h^−1^ compared to the controls in a standard Ringer solution (0.29 ± 0.089 µeq·cm^−2^·h^−1^) (N/n = 5/29; *p* = 0.05), and an expectedly lower G_t_, which was 12.7 ± 0.99 mS·cm^−2^ rather than 21.5 ± 1.08 mS·cm^−2^ (*p* < 0.01). After cinnamaldehyde addition, a ΔI_sc_ response was observed that was numerically smaller than that of the control (0.26 ± 0.056 µeq·cm^−2^·h^−1^ or 53.8 ± 11.5% of the control). The G_t_ increased by ΔG_t_ = 3.42 ± 0.38 mS·cm^−2^, a value that was at 75.5 ± 8.3% of the control (*p* = 0.037).

The tissues that were incubated in parallel in the HCO_3_^−^-free solution (N/n = 5/24) had a baseline I_sc_ of 0.59 ± 0.073 µeq·cm^−2^·h^−1^, which was again significantly higher than that of the controls mentioned above (*p* = 0.014). G_t_ was reduced to 14.1 ± 0.61 mS·cm^−2^ (*p* < 0.01). The response to cinnamaldehyde was strongly reduced, with ΔI_sc_ at 0.11 ± 0.046 µeq·cm^−2^·h^−1^ in this group, or 22.3 ± 9.3% of the control (*p* < 0.001, N/n = 5/29). The ΔG_t_ of 2.90 ± 0.25 mS·cm^−2^ was at 64.2 ± 5.53% of the control (*p* = 0.005).

It is interesting to rank the effects of the removal of the ions on the cinnamaldehyde response in comparison to the effect in a standard Ringer solution using values from all of the controls studied (N/n = 12/70). The values of ΔI_sc_ increased in the order HCO_3_^−^-free < Na^+^-free < low Cl^−^ < standard Ringer < EGTA, with mean values of 0.11 ± 0.046^a^, 0.14 ± 0.039^ab^, 0.26 ± 0.056^b^, 0.63 ± 0.067^c^ and 1.18 ± 0.20^d^ µeq·cm^−2^·h^−1^, respectively, in which the values that do not share a superscript are significantly different. The values of ΔG_t_ ranked in the order Na^+^-free < HCO_3_^−^-free < low Cl^−^ < standard Ringer < EGTA and were 1.16 ± 0.10^a^, 2.90 ± 0.25^b^, 3.42 ± 0.38^bc^, 4.11 ± 0.31^c^, 12.3 ± 1.92^d^ mS·cm^−^^2^.

#### 2.2.4. Effect of Thymol

In further experiments, the response to the herbal diterpene thymol was investigated. Thymol is known as an agonist of TRPM8, TRPV3 and TRPA1. A first set of screening experiments revealed that, as with cinnamaldehyde, thymol only showed effects after the mucosal or bilateral application in the colon, but not after the serosal application (all N/n = 2/4) (see Supplementary Materials Figure S3).

**Figure 5:**
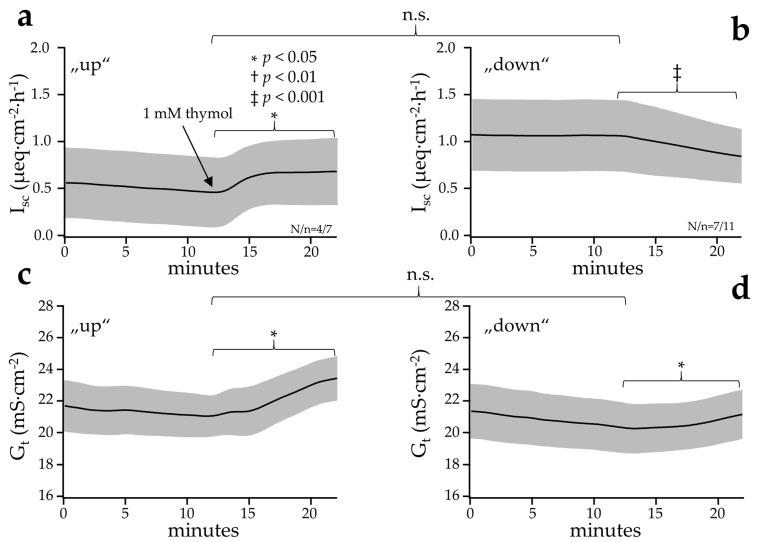
Effect of a bilateral application of thymol on the I_sc_ and G_t_ of the colon of ten young pigs (controlled in-house study). The data are given as means ± SEM. In some of the tissues, an increase (“up“) in I_sc_ could be observed after the addition of thymol (**a**), whereas in other tissues a decrease or no effect (“down“) was observed (**b**). An increase in G_t_ was observed in all of the tissues after the addition of thymol, ruling out barrier effects for increases in I_sc_ (**c**,**d**). The significance bars within graphs compare values taken immediately prior to addition of the agonist and after an incubation of 10 min. The significance bars between graphs indicate that in the colon, there was no difference between the “up” and the “down” groups before thymol was added. Significant differences are marked as *, †, or ‡ (*p* < 0.05, *p* < 0.01 or *p* < 0.001).

The response to the bilateral application of thymol was studied more rigorously in colonic tissues from 10 pigs. As before, a robust rise in G_t_ could be observed in all of the tissues studied (Figure 5, *p* < 0.001 colon). The effects of thymol on the short circuit current I_sc_ were quite variable. In colonic tissues from three pigs, I_sc_ went up; in six other pigs, I_sc_ went down; in one pig, the responses depended on the individual tissue (three down, one up). The means of the tissues that responded to thymol with a pronounced increase in I_sc_ (“up”) are shown in Figure 5a (*p* = 0.016, N/n = 4/7), while the means of the tissues in which I_sc_ dropped (“down”) are shown in Figure 5b (*p* < 0.001, N/n = 7/11).

Similar effects were observed in the jejunum (see Supplementary Materials, Figure S4, N/n = 10/18) and in the caecum (data not shown, N/n = 2/4).

### 2.3. Patch Clamp Studies

The effects of cinnamaldehyde on TRPA1 in overexpressing cells are extremely well documented [1,2]. However, one study has described the fact that cinnamaldehyde can activate TRPV3 in addition to TRPA1 [37], raising questions concerning its specificity. Accordingly, the effect of cinnamaldehyde on TRPV3 was studied using HEK-293 cells transfected with the human variant of TRPV3. As control cells, cells were transfected with the empty vector, essentially as described previously [40,41]. Cells were also treated with thymol, so that a subsequent comparison of the relative response of the TRPV3 expressing cells and the colonic tissues to cinnamaldehyde and thymol was possible.

**Figure 6:**
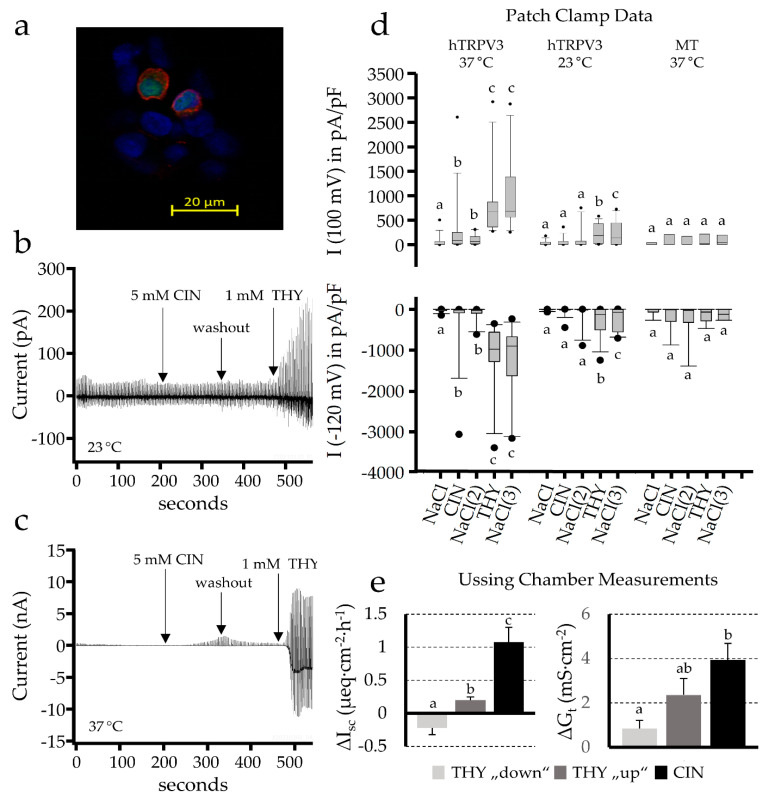
(**a**) Immunohistochemical staining of HEK-293 cells transfected with a vector for the simultaneous overexpression of human TRPV3 and green fluorescent protein (GFP, green), stained with a specific antibody against TRPV3 (red). The cell nuclei were stained with DAPI (blue). (**b**) An original recording of a patch clamp measurement of an hTRPV3 HEK-293 cell at 23 °C. No visible response was seen after the addition of 5 mmol·L^−1^ cinnamaldehyde (CIN), whereas 1 mmol·L^−1^ thymol (THY) elicited a clear response. (**c**) A patch clamp measurement of an hTRPV3 HEK-293 cell at 37 °C. The concentration of cinnamaldehyde had to be elevated to 5 mmol·L^−1^ before a small response could be observed. In contrast, at 1 mmol·L^−1^, the effects of the thymol were very strong. (**d**) A boxplot of patch clamp data from hTRPV3 HEK-293 cells at 37 °C and 23 °C, and from control cells transfected with the empty vector (MT, 37 °C) at +100 mV and −120 mV. Within a group, significant differences after the addition of cinnamaldehyde or thymol and the subsequent washouts (NaCl(2) and NaCl(3)) are indicated via different letters above the bars. Comparisons between the groups are given in the main text (**e**) Data from Ussing chamber experiments from a subset of seven young pigs from Figure 5 (controlled in-house study), treated in parallel with either thymol (N/n = 7/13) or cinnamaldehyde (N/n = 7/14). In the native colonic tissues, 1 mmol·L^−1^ of cinnamaldehyde was sufficient to induce a clear change in the short circuit current and the conductance, which rose by ΔI_sc_ and ΔG_t_, respectively. For comparison, the data for thymol (1 mmol·L^−^^1^) are also shown. Here, the I_sc_ responses were clearly smaller, but diverse, with some tissues showing an increase in I_sc_ (“up”, N/n = 4/7) and others a decrease (“down”, N/n = 4/6). Bars that do not share a letter are significantly different.

The successful transfection of the cells was detected in a first step by the immunohistochemical staining of TRPV3 in the transfected HEK-293 cells. The transfected hTRPV3 cells showed green staining of the cytosol, reflecting co-expression of green fluorescent protein (GFP) as well as a red staining of the cell membrane, demonstrating the successful expression of the TRPV3 channel protein (Figure 6a). The control cells only showed green cytosolic staining (not shown). The transfected hTRPV3 and control cells were then examined with patch-clamp experiments under whole-cell conditions.

In a first series of experiments, cinnamaldehyde was applied to the cells in the concentration used in the Ussing chamber experiments (1 mmol·L^−1^), which did not yield significant effects. In a second series, a higher concentration of 5 mmol·L^−1^ cinnamaldehyde was added to the cells, followed by a washout. At the end of the experiment, the addition of 1 mmol·L^−1^ thymol, which is a strong agonist of TRPV3, served as a control reaction. No response to 5 mmol·L^−1^ cinnamaldehyde was observed in hTRPV3 cells at 23 °C, although these cells reacted strongly to 1 mmol·L^−1^ thymol (Figure 6b). At 37 °C, a slowly increasing current was measured after the addition of cinnamaldehyde (5 mmol·L^−1^), which decreased again after the washout. However, the response was discrete when compared to the response to 1 mmol·L^−1^ thymol (Figure 6c). In contrast, the control cells (also measured at 37 °C) showed no effect from either of the two agonists.

Under the baseline conditions, the three groups of cells (hTRPV3 at 23 °C (N = 8), hTRPV3 at 37 °C (N = 14) and the control at 37 °C (N = 8)) did not show different currents at +100 mV and −120 mV pipette potential, respectively (Figure 6d). No effect was observed in the control group at 37 °C or in the hTRPV3 group at 23 °C. In hTRPV3 at 37 °C, the addition of cinnamaldehyde induced a significant increase in the Na^+^ efflux from the pipette into the bath solution at +100 mV, which rose from 60 ± 35 pA·pF^−1^ to 284 ± 181 pA·pF^−1^ (*p* < 0.001 vs. the baseline and *p* = 0.015 vs. 23 °C), as well as an increase in the Na^+^ influx from the bath solution into the pipette at −120 mV (from −27 ± 11 pA·pF^−1^ to −268 ± 217 pA·pF^−1^, *p* = 0.009 vs. the baseline and *p* = 0.052 vs. 23 °C). After the washout, the currents in the hTRPV3 37 °C group decreased numerically at 100 mV (to 100 ± 31 pA·pF^−1^) and at −120 mV (to −113 ± 54 pA·pF^−1^), but they were still significantly higher than they were initially. In contrast to the 5 mmol·L^−1^ required to induce a response to cinnamaldehyde, a concentration of 1 mmol·L^−1^ thymol caused significant increases in the currents at 100 mV and −120 mV in both hTRPV3 groups, although the current increase was significantly greater in the 37 °C cells (*p* < 0.001 and *p* = 0.002). This difference makes sense because the TRPV3 is activated at warm temperatures (≥32 °C) [42,43,44].

For comparison, the Ussing chamber data of the colonic epithelia from a subset of seven pigs that were treated in parallel in separate chambers with either thymol or with cinnamaldehyde were used. The epithelia that responded to thymol (1 mmol·L^−1^) with an increase in current (N/n = 4/7) were compared to the data from the same pigs treated with cinnamaldehyde (1 mmol·L^−1^) (N/n = 7/14) (Figure 6e). The responsive tissues with a “down” response (N/n = 4/6) to thymol are included in the graph, but they were even smaller.

In summary, the maximal ∆I_sc_ after the addition of cinnamaldehyde (1.08 ± 0.22 µeq·cm^−2^·h^−1^) was significantly higher than after the thymol addition (0.20 ± 0.046 µeq·cm^−2^·h^−1^) (*p* = 0.002). The ∆G_t_, which reflects both the secretion of K^+^ and the absorption of Na^+^ and Ca^2+^, was similar (2.36 ± 0.74 mS·cm^−2^ for 1 mmol·L^−1^ thymol and 3.94 ± 0.75 mS·cm^−2^ for 1 mmol·L^−1^ cinnamaldehyde). In contrast, in the patch clamp experiments on TRPV3 expressing cells, a five-fold higher concentration of 5 mmol·L^−1^ cinnamaldehyde was required to observe a significant response. This response was small and much lower than the response to 1 mmol·L^−1^ thymol. It thus appears that TRPV3 only plays a marginal role, if any, in the response of the colonic epithelium to 1 mmol·L^−1^ cinnamaldehyde.

## 3. Discussion

As outlined in the introduction, TRPA1 agonists are emerging as promising pharmacological tools in the modulation of intestinal function in health and disease [20,32]. Despite this, only a handful of studies have systematically investigated the interaction of TPRA1 agonists with native gastrointestinal epithelia [14,16,39,45].

Because systematic quantitative studies of TRPA1 expression by the gastrointestinal tract seem to be lacking, we wished to find out more about the relative expression of TRPA1 along the porcine gastrointestinal tract via semiquantitative qPCR. In the second part, we studied the electrophysiological effects of the classical and therapeutically promising TRPA1 agonist cinnamaldehyde on epithelia in Ussing chambers. Although some of the experiments were performed on tissues from the porcine jejunum, the primary focus was on the colon as a major locus of fermentation and of inflammatory bowel disease, which also happened to be the tissue with the highest expression of mRNA for TRPA1. Finally, some patch clamp experiments were performed on overexpressing cells in order to assess a possible contribution of TRPV3 to the cinnamaldehyde response [37].

### 3.1. Expression of mRNA for TRPA1 by the Tissues of the Porcine Gastrointestinal Tract

In a first step, semiquantitative PCR was used to investigate the distribution of TRPA1 in various segments of the gastrointestinal tract, namely the fundus and cardia of the stomach, duodenum, jejunum, ileum, caecum, and (middle) colon (Figure 1). The signals for TRPA1 in the muscular layers were below the detection level. With the curious exception of the ileum, the mucosa of all of the sections showed a clear expression of TRPA1, with the expression rising in the distal segments and highest in the colon. This finding is in agreement with immunohistochemical data showing that TRPA1 is expressed along the entire gastrointestinal tract of various species [11,27,39,46].

### 3.2. Effect of Blockers on I_sc_ and G_t_ in Ringer

**Figure 7:**
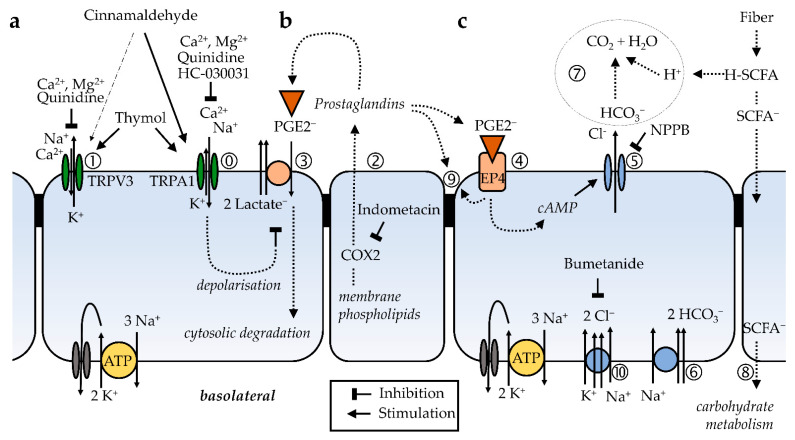
Model of the effects of cinnamaldehyde on the colon, based on the present study and the current literature. (**a**) Cinnamaldehyde opens apical, non-selective TRPA1 channels in the colonic mucosa near the lumen (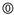). The selectivity filter of the channel allows both the influx of Na^+^ and Ca^2+^, and a smaller efflux of K^+^, so that the effects on I_sc_ are small. However, the cell is depolarised and a significant increase in conductance ΔG_t_ is observed, which is reduced by quinidine and enhanced by the removal of divalent cations. The effects of cinnamaldehyde on TRPV3 (➀), which favors efflux of K^+^ over influx of Na^+^ and Ca^2+^, are discrete. Thymol opens both channels. (**b**) Prostanoids such as PGE2 are anions that are synthesized from membrane phospholipids via cyclooxygenase-mediated pathways and secreted into the extracellular space via pathways that are being explored (➁). For prostanoid signalling to end, the anionic prostaglandin has to be taken up into the cytosol via an electrogenic anion exchanger, OATP2A1 (SLCOA1) (➂), after which the prostaglandin is degraded by cytosolic enzymes. Due to the electrogenic nature of the cotransporter, the depolarization of the cellular membrane, as occurs after the opening of TRPA1 channels via cinnamaldehyde (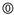), decreases the uptake of prostaglandins and thus increases the extracellular prostaglandin concentration. (**c**) After the binding of the PGE2 to EP4 receptors (➃) expressed by the colonic mucosa, adenylyl cyclase is stimulated, resulting in rising levels of cAMP that open apical CFTR channels (➄). Other anion channels may contribute to the secretion of HCO_3_^−^, which is driven by the uptake of Na^+^ via basolateral NBCn1 (Slc4a7), NBCe1 (SLC4A4), or NBCe2 (Slc4a5) at a ratio of 1, 2 or 3 HCO_3_^−^ for each Na^+^ (➅). Most of the NPPB-sensitive rise in I_sc_ that is observed after the activation of TRPA1 via cinnamaldehyde can be explained by this mechanism. The secretion of HCO_3_^−^ is important for the buffering of protons formed in the fermentational process (➆), and for the unfolding of mucines in the mucus layer, thus protecting the epithelium. Energy-rich short chain fatty acid anions (SCFA^−^) are absorbed via various transport proteins (➇) without challenging cytosolic pH homeostasis. In physiological concentrations, prostaglandins are also thought to have barrier-enhancing properties through interaction with tight junction proteins (➈). Possibly, the secretion of HCO_3_^−^ is highest in cells near the surface, while in the crypts, the expression of NKCC1 (SLC12A2) (➉) predominates. The latter pathway leads to the secretion of Cl^−^ via CFTR, which can result in diarrhea when cAMP levels are pathologically high. Because the gradients favor a unilateral efflux of anions, the opening of CFTR will have higher effects on ΔI_sc_ than those after the opening of TRPA1.

Evidence for the functional expression of TRPA1 by the colon was obtained by applying various blockers to colonic epithelia in Ussing chambers. (Table 1). Quinidine is a highly potent, although unspecific, blocker of numerous cation channels, while HC-030031 is considered to be specific for TRPA1. Both blockers significantly reduced the baseline I_sc_ and G_t_ (Table 1 and Figure 7a). Conversely, the removal of Ca^2+^ can be expected to enhance the permeation of monovalent cations through TRP channels such as TRPA1 and TRPV3 [4]. In line with this, the mucosal replacement of Ca^2+^ with EGTA induced a highly significant increase of baseline G_t_ and I_sc_ (Table 1). Because the experiments were carried out in symmetrical solutions with no chemical gradient present, the rise in I_sc_ clearly reflects transcellular transport, most likely energized by the basolateral Na^+^/K^+^-ATPase. In addition, the removal of Ca^2+^ is known to enhance the permeability of the paracellular pathway by the decoupling of tight junction proteins [47,48], which may explain part of the rise in G_t_.

The effects of the other treatments in Table 1 can be understood with textbook models of colonic transport [49]. The functional expression of ENaC (SCNN1) emerges from the amiloride response, while the effects of bumetanide point toward the basolateral expression of NKCC1, which drives the influx of Cl^−^ for secretion via NPPB-sensitive apical Cl^−^ channels (Figure 7c). These channels close when cAMP production is reduced after the application of the cyclooxygenase inhibitor indometacin, all of which is classically established [49].

### 3.3. Mucosal Cinnamaldehyde Induces an Increase in I_sc_ and G_t_

In a second step, the effect of cinnamaldehyde on stripped epithelium from the jejunum and the colon was investigated in Ussing chambers (Figure 2 and Supplementary Material Figure S1).

Unlike in previous studies of rat colon [16,39] or porcine jejunum [45], the application of cinnamaldehyde in a concentration of 100 µmol·L^−1^ did not result in significant changes in the electrophysiological parameters. This may reflect the particularly thick mucus layer protecting the porcine colon, in conjunction with a partial degradation of cinnamaldehyde by the resident microbials. However, when applied at 1 mmol·L^−1^, significant increases in I_sc_ could be observed in both the colonic (Figure 2) and jejunal epithelia (Supplementary Materials Figure S1). Because, in our study, the colonic and jejunal responses from the same animals were monitored in parallel, they could be directly compared, and it emerged that the responses of the jejunum to cinnamaldehyde were significantly smaller in both ΔG_t_ and ΔI_sc_. It is an attractive hypothesis that this reflects the lower expression of TRPA1 in this segment (Figure 1).

The jejunum responded not only to mucosal or bilateral application but also to the serosal application of cinnamaldehyde. In a previous study of porcine jejunum, the cinnamaldehyde response was inhibited by hexamethonium, but not by TTX. Conversely, the effects of thymol could be completely blocked by TTX [45]. It appears that an interplay of neuronal and epithelial TRP channels regulate the electrophysiological response of the jejunum in a complex manner that we did not attempt to unravel in the present study.

In the colon, the effects were more straightforward because the serosal application of cinnamaldehyde showed no effect (Figure 3). While the serosal application of lidocaine as a blocker of neuronal Na^+^ channels changed the baseline I_sc_ and G_t_ of the colonic epithelium (Table 1), underlining the importance of neuronal signalling for the regulation of transport function, the subsequent response of the tissue to cinnamaldehyde was not altered by pretreatment with lidocaine (Figure 4 and Table 2). Likewise, in previous investigations of rat and human colon, agonists of TRPA1 were most effective when given mucosally, while preincubation with tetrodotoxin did not affect the response of the tissues to AITC, to cinnamaldehyde or to thymol [14,16,39]. In conjunction, these results suggest that the response of the colon to cinnamaldehyde involves mucosal receptors, with TRPA1 channels, as expressed by the apical membrane of human or rat colonocytes [39] being likely candidates.

### 3.4. Does Cinnamaldehyde Activate TRPV3?

Although it is generally considered to be specific for TRPA1, one study has suggested that cinnamaldehyde also opens TRPV3 channels [37]. TRPV3 is expressed by the apical membrane of colonocytes not only in rats and humans [50], but also in the pig (Manneck et al., submitted). Because commercially available specific agonists or inhibitors of TRPV3 are still in the process of being developed [51], we compared the responses of thymol (which strongly activates TRPV3 [25,41]) to those to cinnamaldehyde using Ussing chamber experiments on native epithelia and patch clamp experiments with overexpressing cells.

Previous studies of the thymol response in rat colon have shown a strong rise in I_sc_ and G_t_, resembling the response to cinnamaldehyde, although the signalling differed [14,39]. In porcine colon, the responses to thymol were highly variable, with both increases in I_sc_ and decreases observed, in marked contrast to the uniform responses observed in parallel in tissues from the same pigs after the application of cinnamaldehyde. While changes in the barrier function may have contributed, the selectivity of TRPV3 to Ca^2+^ is poor, and it follows an Eisenman sequence IV with P(K^+^) > P(Na^+^) [4,40], while TRPA1 follows an Eisenman XI sequence with P(Na^+^) > P(K^+^) [1,2]. Accordingly, and depending on the gradients present, the opening of TRPV3 by thymol may lead to a secretion of K^+^ with the hyperpolarization of the apical membrane and a drop in I_sc_. Conversely, the opening of TRPA1 should lead to an increase in I_sc_, as observed with cinnamaldehyde. The different relative expression of TRPA1 or TRPV3 may thus explain the variability of the response to thymol.

In patch clamp experiments overexpressing hTRPV3, as in a previous study of the bovine homologue [41], 1 mmol·L^−1^ thymol elicited the expected large response. However, despite numerous attempts, at 1 mmol·L^−1^, no effect after the application of cinnamaldehyde could be observed. Small effects of cinnamaldehyde were only detectable at 5 mmol·L^−1^ and after the elevation of the bath temperature to 37 °C.

It thus appears that contributions of TRPV3 to the cinnamaldehyde response are possible, but most likely small.

### 3.5. G_t_ Is Sensitive to Quinidine

Further experiments were conducted in order to assess the contribution of cation absorption and/or anion secretion to the current. The lack of an effect of amiloride on the cinnamaldehyde response suggests that ENaC was not involved (Figure 4 and Table 2). Quinidine, a blocker of non-selective cation channels, had a strikingly negative impact on the increase in G_t_ observed after the application of cinnamaldehyde, with ΔG_t_ dropping by more than half (Figure 4), although ΔI_sc_ was not altered. Possibly, the quinidine effects were caused by a previously unknown negative interaction of quinidine with tight junction proteins. However, a more likely hypothesis is that quinidine blocked both the influx of Na^+^ and the efflux of K^+^ through TRP channels such as TRPV3 and TRPA1 by roughly equal amounts (Figure 7a), so that as a net effect, the I_sc_ level remained roughly the same, while the G_t_ dropped to about 40%.

In three different studies of rat colon, the response to AITC, cinnamaldehyde or thymol (100 µmol⋅L^−1^) was significantly reduced by an equivalent concentration of HC-030031 [14,16,39]. On the other hand, the knockout of TRPA1 only partially reduced the AITC response, highlighting the possibility that TRPA1 may not be the only channel involved [16]. In our study, the TRPA1 blocker HC-030031 reduced the cinnamaldehyde response numerically, but the effects did not pass testing for significance (Figure 4 and Table 2). The most likely explanation is that 100 µmol⋅L^−1^ of HC-030031 was insufficient to block the activity of the 1 mmol⋅l^−1^ cinnamaldehyde used in this study.

### 3.6. I_sc_ and ΔG_t_ Can Be Inhibited by Indometacin

The effects of the anion channel blocker NPPB suggest that the cinnamaldehyde-induced rise in I_sc_ is at least partially caused by the opening of an apical anion channel [49] (Figure 7c). Indometacin, which leads to reduced levels of cAMP, had identical effects, pointing towards an involvement of CFTR, although additional anion channels may participate. In our study, neither drug had a significant impact on ΔG_t_. In contrast, in studies of the rat colon, the COX-inhibitor piroxicam reduced both ΔG_t_ and ΔI_sc_ in response to AITC [39].

In the study by Kaji et al. [39], the application of PGE2 induced increases in I_sc_ that could not be further enhanced by the subsequent application of AITC, suggesting that all of the CFTR channels were already at the maximal open probability. Furthermore, in both human and rat colon, the response to AITC could be strongly inhibited by ONO-AE3-208, a specific blocker which prevents the binding of PGE2 to the EP4 receptor.

### 3.7. Bicarbonate Is a Bigger Player Than Chloride in ΔI_sc_ and ΔG_t_

As mentioned above, the basolateral uptake of Cl^−^ classically occurs via NKCC1 (SLC12A2). The responses of the rat and human colon to either thymol or AITC were partially blocked by bumetanide in two previous studies [14,39]. All of the responses could also be significantly reduced by the removal of Cl^−^, with smaller effects after the removal of bicarbonate. Conversely, the cinnamaldehyde response of the pig jejunum was sensitive to the removal of HCO_3_^−^, but insensitive to either chloride removal or bumetanide [45].

In this study, the response of pig colon to cinnamaldehyde resembles the previous findings in pig jejunum [45]. The blocking of NKCC1 by bumetanide did not interfere with the cinnamaldehyde response, showing no significant effect on either ΔI_sc_ or ΔG_t_. Furthermore, the bilateral reduction of Cl^−^ (from 130.3 to 10.3 mmol·L^−1^) had no significant effect on ΔI_sc_, although ΔG_t_ was significantly reduced by about half. Instead, dramatic effects were observed after the removal of only 25 mmol·L^−1^ bicarbonate from the solution via replacement with HEPES and gluconate (Supplementary Materials Table S1). Despite the continued presence of 130.3 mmol·L^−1^ Cl^−^, the cinnamaldehyde-induced ΔI_sc_ dropped to less than a third of the control response, while ΔG_t_ dropped by about 40%. It thus appears that despite having a much lower concentration and only 60% of the mobility (see mobility listings in JPCalcWin 1.01, [52]) HCO_3_^−^ contributes more to the cinnamaldehyde-induced ΔI_sc_ and ΔG_t_ than Cl^−^.

These results are understandable if one assumes that NKCC1 does not contribute much to the cinnamaldehyde response. The basolateral uptake of HCO_3_^−^ most likely occurs via basolateral cotransporters such as NBCe1 (SLC4A4), NBCe2 (Slc4a5) or NBCn1 (Slc4a7), which mediate the cotransport of Na^+^ and HCO_3_^−^, and are amply expressed by the hindgut [53,54,55]. The apical efflux of HCO_3_^−^ should be possible through apical Cl^−^ channels such as CFTR [49,56,57], which, like practically all of the anion channels known to date, are notoriously promiscuous. Either signalling complexes between the NBCs and CFTR or differential expression by distinct cell types may explain the preferential transport of HCO_3_^−^ over Cl^−^. In contrast to the secretion of chloride, the secretion of HCO_3_^−^ should be useful to help with the buffering of short chain fatty acids fermentationally produced from fiber within the colonic lumen [33,34]. This may be of particular importance in pigs, which typically obtain about 30% of their energy from hindgut fermentation [34].

### 3.8. G_t_ Requires the Presence of Mucosal Na^+^

At this point, some deliberations concerning the ΔG_t_ induced by cinnamaldehyde are possible. Given the low selectivity of anion channels, it is very hard to envision a paracellular tight junction protein with a high selectivity for HCO_3_^−^ over Cl^−^. It thus appears that the HCO_3_^−^ dependent fraction, or about 40%, reflect changes in the transcellular passage of HCO_3_^−^. Experiments in low chloride Ringer suggest that 25% of ΔG_t_ reflects the para- or transcellular flux of Cl^−^. In conjunction with the quinidine data, it appears that, in total, roughly half of ΔG_t_ is caused by anions. Despite this, the removal of bilateral Na^+^ had dramatic effects on the cinnamaldehyde response, with ΔI_sc_ and ΔG_t_ at a mere ~ 15 and 20% of the response in the control tissues, respectively. The collapse in ΔI_sc_ is clearly due to the lack of serosal Na^+^ as a driving force for Na^+^-HCO_3_^−^ cotransport. However, if paracellular transport is assumed to be responsible for changes in conductance, the collapse in ΔG_t_ greatly exceeds reasonable expectations. Furthermore, ΔG_t_ collapsed by precisely the same amount when Na^+^ was replaced on the mucosal side only. This observation is not compatible with the assumption of a paracellular flux of Na^+^, because in this case, ΔG_t_ should have been much higher after the unilateral Na^+^ removal than after the bilateral Na^+^ removal. It appears that while a large part of the ΔG_t_ response reflects the passage of anions, the signalling to induce the response occurs via a quinidine-sensitive pathway and requires the presence of mucosal Na^+^. The entry of Na^+^ through TRPA1 is the most likely option.

While the mucosal removal of Na^+^ dramatically reduced ΔG_t_, it had absolutely no effect on the cinnamaldehyde-induced ΔI_sc_. A possible reason for this is that the removal of mucosal Na^+^ decreased the cytosolic Na^+^, thus stimulating the basolateral influx of HCO_3_^−^ via Na^+^-HCO_3_^−^ cotransport and the influx of Cl^−^ via NKCC1, with a subsequent increase in the apical secretion of anions. Another possibility is that the influx of Ca^2+^ was sufficient for the response.

### 3.9. Removal of Ca^2+^ Enhances ΔG_t_

An attractive hypothesis is to assume that the cinnamaldehyde induced increase in I_sc_ is calcium dependent. Rising levels of cytosolic Ca^2+^ typically activate the apical Cl^−^ channels of the colonic epithelium both directly (in the case of calcium-dependent Cl^−^ channels) and indirectly (via calcium-dependent adenylyl cyclases with the production of cAMP) [49,58]. Thus, in rat colon, the thymol-induced ΔI_sc_ was reduced in bilateral Ca^2+^-free Ringer, although notably, the ΔG_t_ remained the same [14]. In contrast, Ca^2+^ removal did not affect the AITC response of rat colon in a study by the same authors [39].

In the current study of porcine colon, bilateral nominally Ca^2+^-free solution did not lead to a reduction in the cinnamaldehyde-induced ΔI_sc_. Instead, the cinnamaldehyde-induced ΔG_t_ rose to almost twice the size observed in controls from the same animals. When Ca^2+^ was replaced by EGTA on the mucosal side only, ΔG_t_ rose even further to a striking 300% of the controls (*p* < 0.001). Simultaneously, ΔI_sc_ rose numerically to 150% of the controls from the same animals, a result that tested for significance when compared to the entire set of the controls. It thus appears that Ca^2+^ is very clearly not necessary for the response of the tissues to cinnamaldehyde.

### 3.10. Does the Opening of TRPA1 Inhibit the Uptake and Degradation of Prostaglandins?

While further work is clearly necessary, some speculation is possible. The synthesis of PGE2 by the colon is well-documented, and the effects of the inhibition of prostaglandin synthesis were significant and seen not only in this study, but also in two separate studies of rat colon using AITC as a TRPA1 agonist [16,39]. Prostanoids such as PGE2 are anions that are synthesized from membrane phospholipids via cyclooxygenase-mediated pathways. After secretion into the extracellular space, prostanoids are bound to specific prostanoid receptors [59,60]. For prostanoid signalling to end, the anionic prostaglandin has to be taken up into the cytosol where it is degraded. This uptake occurs via an electrogenic anion exchanger, OATP2A1 (SLCO2A1), with the efflux of two lactate anions driving the influx of one prostaglandin anion. Accordingly, the depolarization of the cellular membrane decreases the uptake of PGE2 [59,60,61]. The events are thus as follows: PGE2 is continuously produced by the colonic epithelium and taken back up into the cell via OATP2A1. If TRPA1 is opened via cinnamaldehyde or AITC, the entry of Na^+^ and Ca^2+^ will exceed the efflux of K^+^, depolarizing the cell. This reduces the reuptake of PGE2 via OATP2A1. There is thus more PGE2 to bind to EP4, leading to the activation of adenyl cyclase, the production of cAMP, the opening of CFTR and, finally, the secretion of HCO_3_^−^ and Cl^−^. In conjunction, a rise in the current (ΔI_sc_) and a rise in conductance (ΔG_t_) are observed.

### 3.11. Barrier Effects

As outlined above, we do not think that an opening of the paracellular pathway can explain the major part of the cinnamaldehyde-induced ΔG_t_ response. From this study and others, there is considerable evidence to suggest that the activation of TRPA1 induces the secretion of PGE2 [16,39]. PGE2 has direct barrier-enhancing properties [62,63]. Thus, in cell culture models of colonic epithelia, prostaglandin-mediated signalling via the EP2 receptor prevented the proteosomal degradation of Claudin-4 [64]. The colon of EP4 deficient mice showed increased rates of apoptotic cells, as well as a defective mucosal barrier with signs of inflammation [65]. Furthermore, PGE2 was found to stimulate the recovery of the barrier function in porcine ischemia-injured ileal mucosa [66]. In addition, lower levels of PGE2 and other prostaglandins are thought to be involved in the classical gastrointestinal side-effects of cyclooxygenase inhibitors [67]. However, it is important to bear in mind that the pharmacology of PGE2 is notoriously complex, with numerous pro- and anti-inflammatory effects that are probably related to local concentrations of PGE2 and the expression patterns of its various receptors [62].

### 3.12. Cinnamaldehyde, Bicarbonate and the Buffering of Fermentational Acids in the Colon

More work is clearly necessary to understand the interaction of essential oils in general and of cinnamaldehyde in particular with colonic epithelia, but a picture is slowly emerging (Figure 7). In humans and in animals, the function of the colon is to serve as a fermentation chamber in which microbials can degrade the structural carbohydrates that are resistant to enzymatic digestion of the small intestine [33,34]. Anaerobic fermentation produces large quantities of short-chain fatty acids (Figure 7c). Large quantities of protons are released via dissociation and have to be removed, since low values of pH produce shifts in the colonic microbiome towards species that produce lactic acid. This is an occurrence associated with a further drop in colonic pH and subsequent damage to the epithelium [38,68,69]. The secretion of bicarbonate with the subsequent formation of CO_2_ is a highly appropriate and well-established buffering mechanism [53,54,57]. While protons are removed via the formation of CO_2_, the anions of short-chain fatty acids can then be transported across the epithelium without impairing the cellular pH homeostasis via various pathways that have been discussed elsewhere [33]. In a further twist, the secretion of HCO_3_^−^ enhances the unfolding of the mucines that protect the epithelium from the bacterial invasion and inflammation seen in inflammatory bowel disease [38,70].

The current study suggests that the ingestion of plants rich in terpenes—such as those contained in the bark of the *Cinnamomum verum* tree—may help to stimulate HCO_3_^−^ secretion. After the degradation of plant structures by microbials in the colon, cinnamaldehyde is released and binds to TRPA1, preventing the cellular uptake and degradation of PGE2 (Figure 7a,b). The rising levels of cAMP will then stimulate the secretion of HCO_3_^−^ (Figure 7c). The removal of protons should certainly help to prevent damage to the epithelial cells, and may explain part of the anti-inflammatory action of essential oils such as cinnamaldehyde. Of course, the amounts consumed must not be too high, because otherwise the secretion of chloride might lead to diarrhea and other disagreeable or even toxic effects. It appears possible that humans and animals alike will use their outstanding ability to detect the smallest quantities of essential oils via their sense of smell to precisely assess just how much is needed to make the food tasty, but not too spicy [71].

## 4. Materials and Methods

### 4.1. Gastrointestinal Tissue

The porcine gastrointestinal tissues were obtained according to the guidelines of the German Animal Welfare Law under oversight by the local authority of the “Landesamt für Gesundheit und Soziales Berlin” (LaGeSo Reg. Nr. T0264/15 and T0297/17). The pigs were a cross between the Danbred x Piétrain breeds, weighing ~25 kg and aged about 10 weeks, and were fed a normal diet. The animals were killed by prior sedation with ketamine (Ursotamin^®^, Serumwerk Bernburg AG, Bernburg, Germany) and azaperone (Stresnil^®^, Jansen-Cilag, Neuss, Germany) by intramuscular injection, followed by an intracardiac injection of tetracaine hydrochloride, mebezonium iodide and embutramide (T61^®^, Intervet Deutschland GmbH, Unterschleissheim, Germany). In a few experiments, older pigs from a commercial slaughterhouse were used, which is indicated where applicable. After death, the gastrointestinal tissue was immediately removed.

### 4.2. Molecular Detection of the TRPA1 Channel in the Gastrointestinal Tissue

For the molecular investigations, the removed gastrointestinal tissue (the fundus and cardia of the stomach, duodenum, mid-jejunum, ileum, caecum and mid-colon) was thoroughly rinsed with PBS, and small pieces of 1 cm^3^ of the tunica muscularis or the mucosa were transferred into tubes containing 1 mL of RNAlater^®^ (Sigma-Aldrich, Taufkirchen, Germany). These were cooled at 4 °C overnight and then stored at −80 °C. For the RNA isolation, a Nucleospin RNA II kit (Macherey-Nagel, Dueren, Germany) was used, and the RNA integrity numbers (RIN) were determined using an RNA 600 Nano kit (Agilent, Waldbronn, Germany). The samples from 4 pigs (out of 6) with the best RIN values (RIN > 6.8 for the ileal epithelium and RIN > 7.3 for all of the other tissues) were subsequently processed. For the reverse transcription into cDNA, an iScript^®^ cDNA synthesis kit (Bio-Rad Laboratories, Munich, Germany) was used according to the manufacturer’s instructions, whereby 1 µg RNA was transcribed per sample and then diluted 1:10.

Afterwards, an exon spanning FAM/BHQ1 labelled primer-probe combination was designed according to the predicted sequence of the porcine TRPA1 channel, and the corresponding reference genes were established (Table 3), which were synthesized by Eurofins (Eurofins Genomics Germany GmbH, Ebersberg, Germany). In order to ensure that the correct target was bound, the amplification product was sequenced and compared to the target sequence (Eurofins Genomics Germany GmbH, Ebersberg, Germany). Primer–probe combinations were also used for the three selected reference genes: ACTB (FAM/BHQ1), GAPDH [72] and YWHAZ (FAM/TAMRA). For the semi-quantitative analysis by qPCR, a 40-cycle 2-step protocol (1 s 95 °C, 20 s 60 °C) was performed on a thermocycler (ViiA 7, Applied Biosystems/Life Technologies, Waltham, MA, USA). The reactions were performed in triplicates with 3.7 µL cDNA and iTaq^®^ Universal Probes Supermix (Bio-Rad Laboratories GmbH, Feldkirchen, Germany) with a total volume of 10 µL. Negative controls (no template controls) were routinely included. The quantification cycles (C_q_) were calculated automatically by the cycler software. The dilution series-based gene-specific amplification efficiencies for the primer pairs were determined and the reference genes were tested for their expression stability (qbasePLUS, Biogazelle NV, Zwijnaarde, Belgium). The C_q_ values of the target gene TRPA1 were normalized using ACTB, GAPDH, and YWHAZ, and were scaled to the sample means. Subsequently, the values were exported as calibrated normalized relative quantity (CNRQ) values using qbasePLUS.

Attempts to find a suitable antibody against TRPA1 were unfortunately unsuccessful. Five commercial antibodies were tested, but yielded multiple or no bands in immunoblots of porcine tissues (sc-166469 and sc-376495, Santa Cruz Biotechnology Inc, Dallas, TX, USA; ACC-037, Alomone, Jerusalem, Israel; TA338564, OriGene, Herford, Germany and AG1346, Abgent, San Diego, CA, USA).

### 4.3. Ussing Chamber Studies

For the electrophysiological studies using a Ussing chamber, the tissue was washed with transport buffer after the removal. The tunica muscularis was stripped, and the tissue was transported with ice-cooled gassed (95% O_2_/5% CO_2_) transport buffer (in mmol·L^−1^: 115 NaCl, 0.4 NaH_2_PO_4_, 2.4 Na_2_HPO_4_, 5 KCl, 25 NaHCO_3_, 5 glucose, 10 HEPES, 1.2 CaCl_2_, 1.2 MgCl_2_). The tissue was then mounted in 0.95 cm^2^ classical, custom-built Ussing chambers with perfusion maintained via a gas-lift system [73,74], which were filled with 10 mL Ringer’s solution (in mmol·L^−1^: 120 NaCl, 25 NaHCO_3_, 0.32 NaH_2_PO_4_, 1 MgSO_4_, 6.3 KCl, 2 CaCl_2_) per side, unless otherwise declared. In order to exclude a glucose-induced sodium current, 16 mmol·L^−1^ glucose was added to the serosal side and 16 mmol·L^−1^ mannitol was added to the mucosal side. The final osmolality of the solutions was adjusted to 300 mosmol·kg^−1^ with mannitol. During the experiment, the tissue was kept permanently at 37 °C and gassed with 95% O_2_ and 5% CO_2_, whereas the solutions without HCO_3_^−^ were gassed with oxygen. After the tissue mounting, the measurements were performed in short-circuit mode and the current (I_sc_) was recorded, with positive values reflecting the transport of cations from the mucosal to the serosal side. Throughout, the I_sc_ represents the molar flux (in µeq·cm^−2^·h^−1^), which can be calculated from the current Φt (in µA·cm^−2^) according to I_sc_ = Φt/F·3600s·h^−1^ = Φt/26.80 µeq·cm^−^^2^·h^−1^, where F is the Faraday constant. Using a 100 µA current pulse and the corresponding potential response, the conductance (G_t_, in mS·cm^−2^) was continuously recorded (Mussler Scientific Instruments, Aachen, Germany).

Measurements commenced after the I_sc_ and G_t_ values had stabilized, or after a maximum of 45 min. All of the agonists were added directly via a pipette to the bath solution in a ratio of 1:1000 to yield the target concentration, with the substances dissolved in either water (amiloride), ethanol (cinnamaldehyde, thymol, lidocaine, bumetanide, indometacin) or dimethyl sulfoxide (DMSO) (quinidine, 5-nitro-2-(3-phenylpropyl-amino) benzoic acid (NPPB), HC-030031). Cinnamaldehyde (1 mmol⋅L^−1^) was added 15 min later. In the other experiments, the solution changes occurred 15 min before the addition of cinnamaldehyde. The composition of the solutions used can be found in the Supplementary Materials Table S1.

### 4.4. Patch Clamp Studies

A human construct of TRPV3 (hTRPV3) was used for the patch-clamp experiments, essentially as in [40,41]. The sequence (GeneArt, Thermo Fisher Scientific, Regensburg, Germany) was tagged with hemagglutinin (HA) and streptavidin (Strep). This construct was subsequently subcloned into pIRES2-AcGFP1 (Takara BioEurope, Saing-Germain-en-Laye, France). HEK-293 cells were used for transient transfection (DSMZ, Braunschweig, Germany). The cells were cultured using Dulbecco’s modified Eagle’s medium supplemented with 10% fetal bovine serum and 100 units·mL^−1^ of penicillin and streptomycin (Biochrom, Berlin, Germany). Approximately 24 h before the start of the experiment, the cells were transfected with polyethyleneimine and the HA-Strep-hTRPV3-pIRES-AcGFP1 construct or the empty pIRES-AcGFP1 vector (control). Accordingly, the successfully transfected hTRPV3 cells should show green fluorescence. Furthermore, the cells were stained with a mouse TRPV3 antibody (ABIN863127, antibodies-online GmbH, Aachen, Germany) at a 1:1000 dilution, as in [40].

The whole-cell experiments were performed at room temperature (adjusted to 23 °C with an airconditioning system) and at 37 °C, using an inline solution heater to adjust the temperature (PH01 (S/N 1007), Multichannel Systems). Patchmaster software (HEKA Electronic) automatically performed the generation of pulses, data collection, filtering with a 2.9 kHz Bessel filter, and correction for capacity and series resistance. A low sampling rate (100 Hz) pulse protocol was used for the recording in order to assess the solution changes. This automatically switched to a classical step protocol with a high sampling rate (5 kHz) to assess the channel kinetics, as in previous studies [40,41]. After the measuring the osmolality, the solutions were adjusted to 300 mosmol·kg^−1^ with mannitol and buffered to a pH of 7.4. The pipette solution contained (in mmol·L^1^): 5 CsCl, 6.63 NaCl, 127.36 Na-gluconate, 10 EGTA, 10 HEPES, 1.91 CaCl_2_, 2.27 MgCl_2_, 1 Mg-ATP. The extracellular solution contained (in mmol·L^−1^): 5 KCl, 1 NaH_2_PO_4_, 137 NaCl, 10 HEPES, 1.7 CaCl_2_, 0.9 MgCl_2_, 5 glucose. In the experiment, the effect of 1 and 5 mmol·L^−1^ cinnamaldehyde was studied at 37 °C and 23 °C, with 1 mmol·L^−1^ thymol serving as a control reaction at the end of the experiment.

### 4.5. Data and Statistics

The statistical analysis was performed with the program SigmaPlot 11.0 (Systat Software, Erkrath, Germany), with the data being tested for normal distribution (Shapiro-Wilk) and homogeneity of variances (Brown-Forsythe). Subsequently, data were tested using a parametric test (Student’s t-test or ANOVA (Student-Newman-Keuls or Dunn’s method)) or a non-parametric test (Mann-Whitney U test or ANOVA on ranks (Kruskal-Wallis method)), as appropriate. Statistical significance was assumed at *p* < 0.05. The data are presented as the mean values ± SEM. The number of experiments with pigs is expressed as N/n, where N is the number of animals and n is the number of tissues. The Ussing chamber data were binomially smoothed using Igor Pro 6.37 (WaveMetrics Inc., Lake Oswego, OR, USA). The statistical analysis of the qPCR data was performed using the calculated CNRQ data (qbasePLUS, Biogazelle NV, Zwijnaarde, Belgium). In the barplots, different letters were placed above the bars to designate significant differences. Bars that do not share a letter are significantly different (*p* < 0.05). Conversely, bars that share at least one letter are not different (*p* > 0.05).

## Figures and Tables

**Table 1:** Baseline I_sc_ (µeq·cm^−2^·h^−1^) and G_t_ (mS·cm^−2^) in colonic tissue from young pigs (controlled in-house study) (baseline) and the peak value in the 15 min interval after the addition of blockers or ion replacement (treatment). For the concentrations and solutions, see Results and Supplementary Materials.

Treatment	N/n ^1^	I_sc_ Baseline	I_sc_ Treatment	*p*-Value	G_t_ Baseline	G_t_ Treatment	*p*-Value
Control (all)	12/70	0.38 ± 0.056	0.37 ± 0.056	0.094	20.5 ± 0.62	20.3 ± 0.61	<0.001
Quinidine	4/21	0.39 ± 0.088	0.23 ± 0.085	<0.001	22.5 ± 1.02	19.9 ± 0.83	<0.001
Lidocaine	3/12	0.41 ± 0.070	0.27 ± 0.069	<0.001	23.6 ± 1.37	22.8 ± 1.10	0.151
Amiloride	3/15	0.55 ± 0.16	0.29 ± 0.15	<0.001	23.0 ± 1.65	21.3 ± 1.69	<0.001
Na^+^ free bilat ^2^	3/17	0.42 ± 0.066	−0.40 ± 0.052	<0.001	18.2 ± 1.58	8.9 ± 0.53	<0.001
Na^+^ free muc ^3^	5/28	0.34 ± 0.044	−0.69 ± 0.062	<0.001	17.6 ± 0.95	12.2 ± 0.39	<0.001
EGTA muc ^2^	4/17	0.77 ± 0.16	0.95 ± 0.21	0.01	23.8 ± 1.38	28.5 ± 1.86	<0.001
Ca^2+^ free bilat ^3^	5/14	0.98 ± 0.068	0.99 ± 0.065	0.819	19.3 ± 0.96	20.3 ± 0.86	0.011
Bumetanide	3/15	0.30 ± 0.11	0.19 ± 0.10	<0.001	20.1 ± 0.94	20.2 ± 0.90	0.528
NPPB	5/21	0.46 ± 0.10	0.26 ± 0.083	<0.001	21.9 ± 1.45	22.8 ± 1.53	0.008
Indometacin	3/9	0.24 ± 0.049	0.16 ± 0.036	0.04	22.6 ± 1.29	22.9 ± 1.39	0.212
Cl^−^ low ^4^	5/30	0.29 ± 0.089	0.52 ± 0.071	0.050	21.5 ± 1.08	12.7 ± 0.99	<0.001
HCO_3_^−^ free ^4^	5/24	0.29 ± 0.089	0.59 ± 0.073	0.014	21.5 ± 1.08	14.1 ± 0.61	<0.001
HC-030031	3/7	0.33 ± 0.038	0.27 ± 0.028	0.028	17.7 ± 1.02	17.9 ± 1.15	0.813

^1^ number of animals/number of tissues; ^2^ bilat = bilateral; ^3^ muc = mucosal; ^4^ test vs. control tissue.

**Table 2:** Cinnamaldehyde response in the colonic tissue from young pigs (controlled in-house study). The tissues were pretreated as indicated in the first column, yielding the values “I_sc_ treatment” (µeq·cm^−2^·h^−1^) and “G_t_ treatment” (mS·cm^−2^) from Table 1. Subsequently, 1 mmol·L^−1^ cinnamaldehyde was added. The columns “I_sc_ cinn.” (µeq·cm^−2^·h^−1^) and “G_t_ cinn.” (mS·cm^−2^) designate the peak value of the responses within a 15 min interval after the application of cinnamaldehyde.

Treatment	N/n ^1^	I_sc_ Treatment	I_sc_ Cinn.	*p*-Value	G_t_ Treatment	G_t_ Cinn.	*p*-Value
Control (all)	12/70	0.38 ± 0.056	1.01 ± 0.10	<0.001	20.3 ± 0.61	24.4 ± 0.79	<0.001
Quinidine	4/21	0.23 ± 0.085	1.19 ± 0.21	<0.001	19.9 ± 0.83	21.7 ± 0.92	<0.001
Lidocaine	3/12	0.27 ± 0.069	1.05 ± 0.22	<0.001	22.8 ± 1.10	29.2 ± 1.44	<0.001
Amiloride	3/15	0.29 ± 0.15	1.29 ± 0.28	<0.001	21.3 ± 1.69	25.0 ± 1.96	<0.001
Na^+^ free bilat ^2^	3/17	−0.40 ± 0.052	−0.26 ± 0.060	0.002	8.90 ± 0.53	10.1 ± 0.58	<0.001
Na^+^ free muc ^3^	5/28	−0.69 ± 0.062	−0.19 ± 0.083	<0.001	12.2 ± 0.39	13.3 ± 0.43	<0.001
EGTA muc ^2^	4/17	0.95 ± 0.21	2.13 ± 0.26	<0.001	28.5 ± 1.86	40.8 ± 3.36	<0.001
Ca^2+^ free bilat ^3^	5/14	0.99 ± 0.065	1.78 ± 0.084	<0.001	20.3 ± 0.86	26.7 ± 1.56	<0.001
Bumetanide	3/15	0.19 ± 0.10	1.16 ± 0.27	<0.001	20.2 ± 0.90	25.2 ± 1.29	<0.001
NPPB	5/21	0.26 ± 0.083	0.71 ± 0.12	<0.001	22.8 ± 1.53	28.6 ± 2.19	<0.001
Indometacin	3/9	0.16 ± 0.036	0.54 ± 0.099	0.004	22.9 ± 1.39	30.6 ± 2.05	<0.001
Cl^−^ low ^3^	5/30	0.52 ± 0.071	0.78 ± 0.10	<0.001	12.7 ± 0.99	16.1 ± 1.28	<0.001
HCO_3_^−^ free ^3^	5/24	0.59 ± 0.073	0.70 ± 0.11	0.054	14.1 ± 0.61	17.1 ± 0.61	<0.001
HC-030031	3/7	0.27 ± 0.028	0.91 ± 0.16	0.004	17.9 ± 1.15	22.3 ± 2.15	0.007

^1^ number of animals/number of tissues; ^2^ bilat = bilateral; ^3^ muc = mucosal.

**Table 3:** Primer sequences and the amplicon length of the genes used.

Gene	Length (bp)	Primer	Gene Acession No.
TRPA1 fwd	192	ACAGGAAAGTCAGCCCTCTC	XM_001926115.4
TRPA1 rev		TATCCTGGCTGCCCGAATAG	
TRPA1 probe		TTTGCGGCCACCCAGGGAGC	
ACTB fwd	127	GACATCAAGGAGAAGCTGTG	XM_003124280.5
ACTB rev		CGTTGCCGATGGTGATG	
ACTB probe		CTGGACTTCGAGCAGGAGATGGCC	
YWHAZ fwd	113	AAGAGTCATACAAAGACAGCAC	XM_021088756.1
YWHAZ rev		ATTTTCCCCTCCTTCTCCTG	
YWHAZ probe		ATCGGATACCCAAGGAGATGAAGCTGAA	
GAPDH fwd	117	CAAGAAGGTGGTGAAGCAG	NM_001206359.1
GAPDH rev		GCATCAAAAGTGGAAGAGTGAG	
GAPDH probe		TGAGGACCAGGTTGTGTCCTGTGACTTCAA	

## Data Availability

Data available on request.

## Supplementary Materials

The following are available online at https://www.mdpi.com/article/10.3390/ijms22105198/s1, Figure S1: Concentration effects of cinnamaldehyde in the jejunum; Figure S2: Effect of cinnamaldehyde after the mucosal, serosal and bilateral addition in the jejunum; Figure S3: Effect of thymol after the mucosal, serosal and bilateral addition in the colon; Figure S4: Effect of the bilateral addition of thymol in the jejunum; Table S1: Ussing chamber solutions.
