# Supplementary material for: The TRPA1 Agonist Cinnamaldehyde Induces the Secretion of HCO3− by the Porcine Colon
Source: Int J Mol Sci. 2021 May 14;22(10):5198. doi: 10.3390/ijms22105198 (PMC8156935; doi:10.3390/ijms22105198)
Supplement: Supplementary file 1 [file ijms-22-05198-s001.zip › 20210513_Supplementary_Materials.pdf]

Supplementary Materials

The TRPA1 Agonist Cinnamaldehyde Induces Secretion of HCO_3^- by the Porcine Colon (Supplement)

David Manneck [1], Gisela Manz [1], Hannah-Sophie Braun [2], Julia Rosendahl [2] and Friederike Stumpff [1,*]

[1] Institute of Veterinary Physiology, Department of Veterinary Medicine, Freie Universität Berlin, Oertzenweg 19b, 14163 Berlin, Germany; david.manneck@fu-berlin.de (D.M.); gisela.manz@fu-berlin.de (G.M.)

[2] PerformaNat GmbH, Hohentwielsteig 6, 14163 Berlin, Germany; hannah-sophie.braun@fu-berlin.de (H.-S.B.); rosendahl@performanat.de (J.R.)

* Correspondence: stumpff@zedat.fu-berlin.de; Tel.: +49-30-838-62595

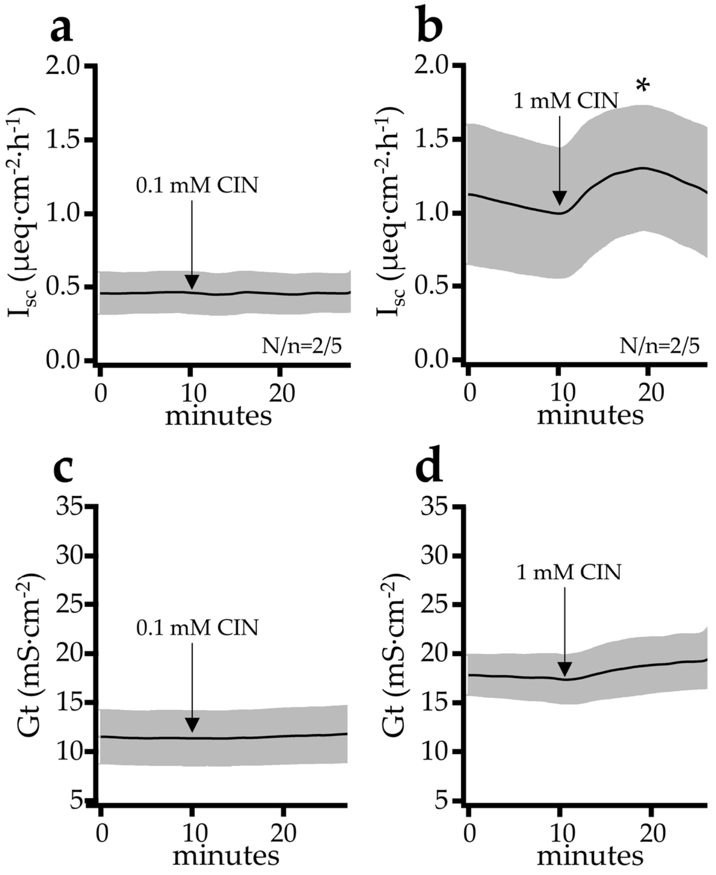

Figure 1. Effect of the TRPA1 agonist cinnamaldehyde at a concentration of 100 µmol·l^{-1} (**a,c**) and 1 mmol·l^{-1} (**b,d**) on I_{sc} and G_t in the Ussing chamber using jejunal tissue of pigs. After the addition of 1 mmol·l^{-1} cinnamaldehyde, a significant increase on the I_{sc} and a trend for G_t could be observed, which was absent when a concentration of 100 µmol·l^{-1} was added. Significant differences are marked as * ($P < 0.05$). N/n = number of animals/number of tissues, identical for G_t and I_{sc}.

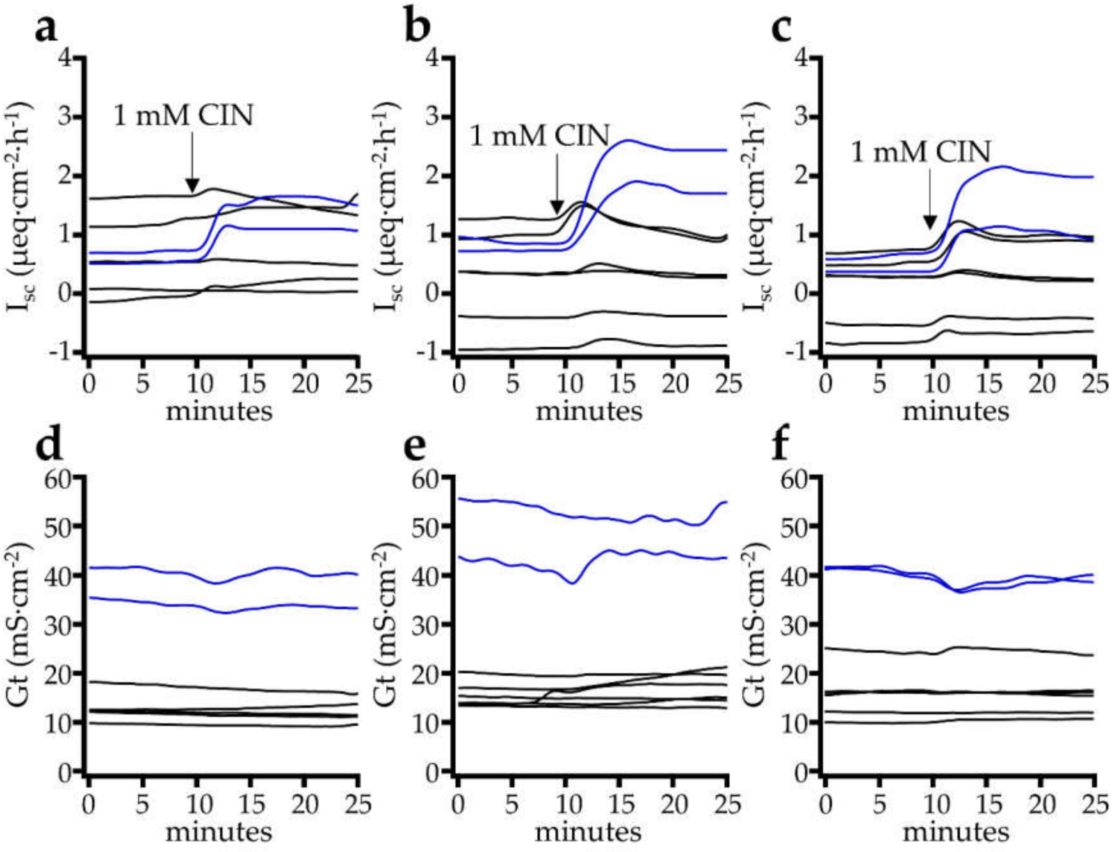

Figure S2. Effect of the TRPA1 agonist cinnamaldehyde (CIN) at a concentration of 1 mmol•l^{-1} after mucosal (**a, d**), serosal (**b, e**), or bilateral addition (**c, f**) on I_{sc} and G_t in the Ussing chamber using jejunal tissue of pigs. After addition, an increase in I_{sc} was observed after mucosal (P = 0.054), serosal (P = 0.008) and after bilateral addition (P = 0.011). However, G_t barely changed after addition in all 3 groups and was not significantly altered. Some tissues were obtained from older pigs from a commercial slaughterhouse (blue lines), while the black lines reflect responses of younger pigs slaughtered within a controlled study as in the rest of the manuscript.

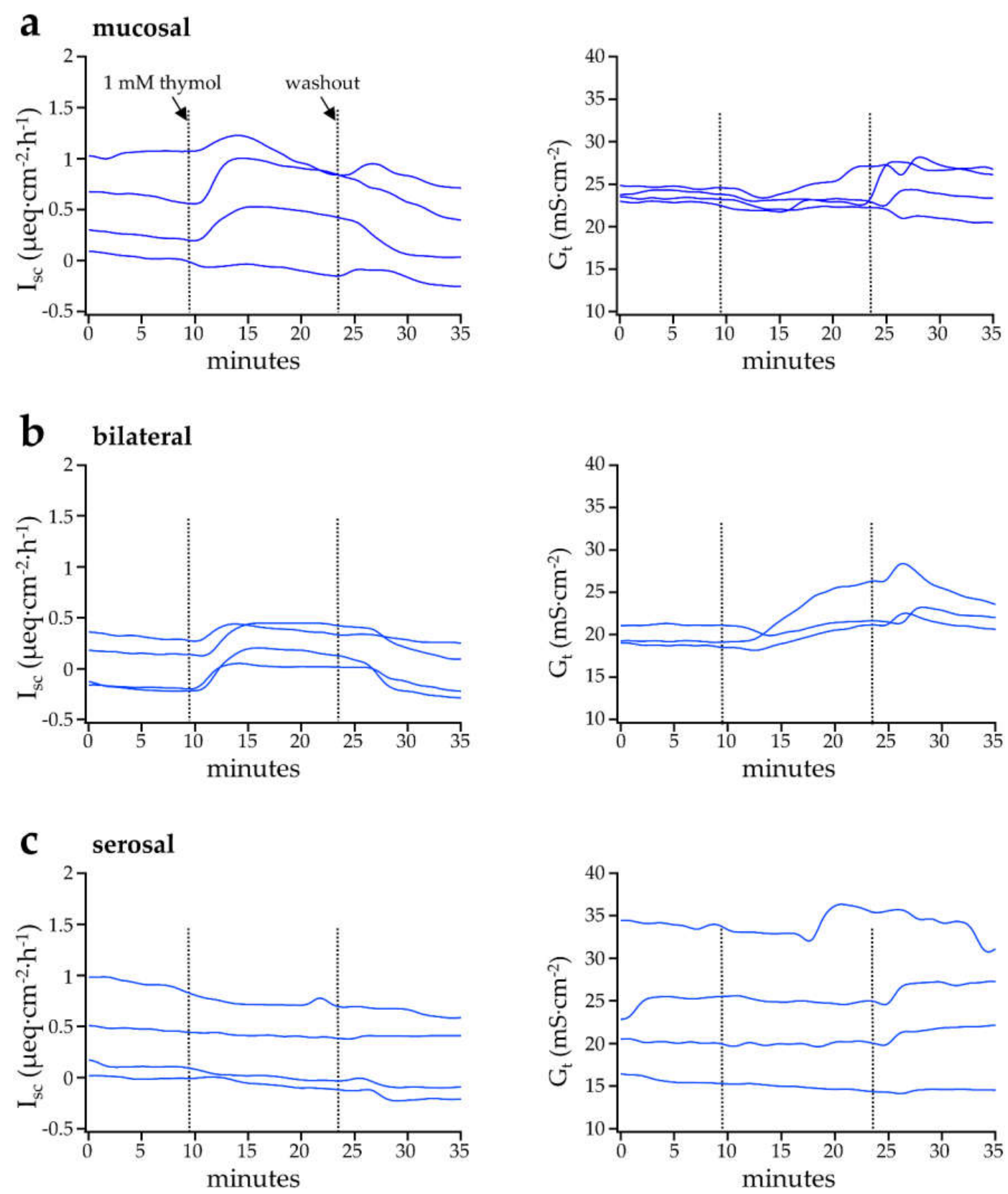

Figure S3. Effect of thymol at a concentration of 1 mmol•l^{-1} after mucosal (**a**), bilateral (**b**), or serosal addition (**c**) on I_{sc} and G_t in the Ussing chamber using colonic tissue of pigs. After mucosal and bilateral addition, a significant increase in I_{sc} and G_t was observed in most tissues, although the effects were absent with serosal addition.

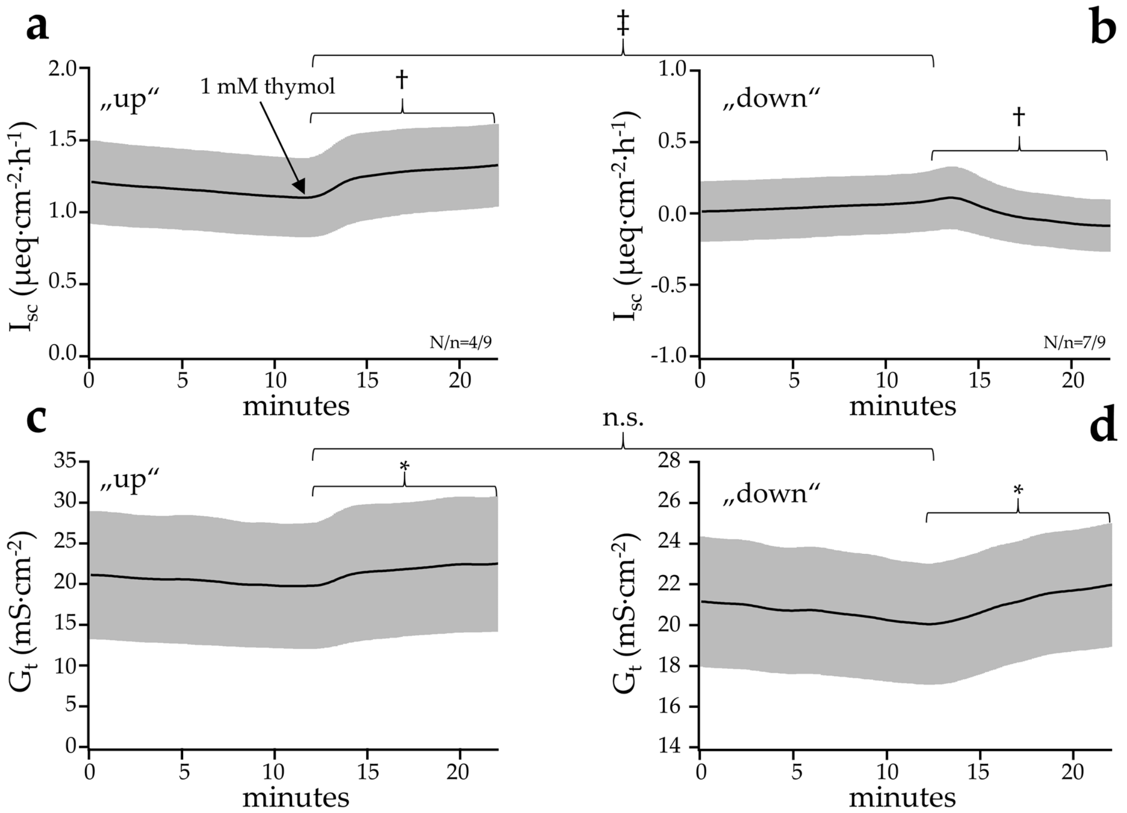

Figure S4. Effect of a bilateral application of thymol on the I_{sc} and G_t of the jejunum of pigs. Data are given as means ± SEM. In some tissues an increase („up“) in I_{sc} could be observed after thymol addition (**a**), whereas in other tissues a decrease or no effect („down“) was observed (**b**). An increase in G_t was observed in all tissues after thymol addition, ruling out barrier effects as a cause for increases in I_{sc} (**c, d**). The significance bars within graphs compare values taken immediately prior to addition of the agonist and after an incubation time of 10 minutes. The significance bars between graphs indicate that in the jejunum, the I_{sc} values were higher in the "up" group from the start. Significant differences are marked as *, †, or ‡ ($P < 0.05$, $P < 0.01$ or $P < 0.001$). N/n = number of animals/number of tissues, identical for G_t and I_{sc}

Table 1. Solutions used in the Ussing chamber experiments. Mucosal and serosal solutions differed only by the addition of 16 mmol·l⁻¹ glucose (serosal) and mannitol (mucosal), respectively, unless otherwise indicated. Solutions were adjusted to 300 mosmol·kg⁻¹ with mannitol and were titrated to a pH of 7.4 (Tris, HCl).

Item (mmol·l⁻¹)	Standard	Na^+ free[1]	Cl^- low	HCO_3^- free	Ca^{2+} free	EGTA[2]
NaCl	120	0	0	120	120	120
$NaHCO_3$	25	0	25		25	25
NaH_2PO_4	0.32	0	0.32	0.32	0.32	0.32
$MgSO_4$	1	1	1	1	1	1.15
KCl	6.3	5.98	6.3	6.3	2	6.3
$CaCl_2$	2	2	2	2	0	0
EGTA	0	0	0	0	0	1
NMDGCl	0	120.32	0	0	0	4
Choline-HCO_3	0	25	0	0	0	0
KH_2PO_4	0	0.32	0	0	0	0
NaGlu	0	0	120	25	0	0
HEPES	0	0	0	10	0	0

[1] Na+ free solution was used as mucosal free only or bilateral free solution. [2] The Ca^{2+} free solution with EGTA was used only on the mucosal side, the serosal side contained the standard solution.
