# Supplementary figures and images for: The TRPA1 Agonist Cinnamaldehyde Induces the Secretion of HCO3− by the Porcine Colon
Source: Int J Mol Sci. 2021 May 14;22(10):5198. doi: 10.3390/ijms22105198 (PMC8156935; doi:10.3390/ijms22105198)

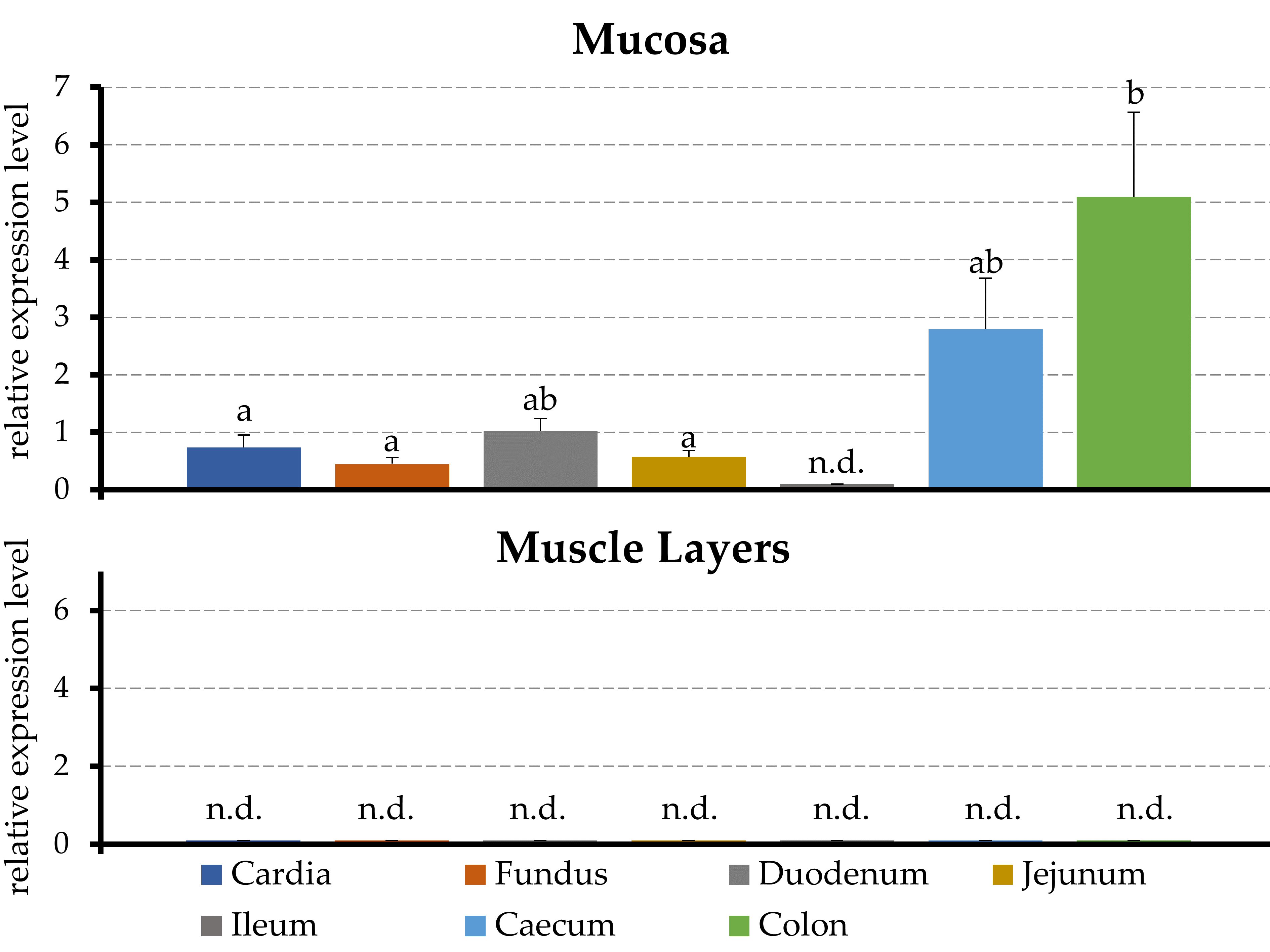

Supplement: Supplementary file 1 [file ijms-22-05198-s001.zip › Figure1 .png]

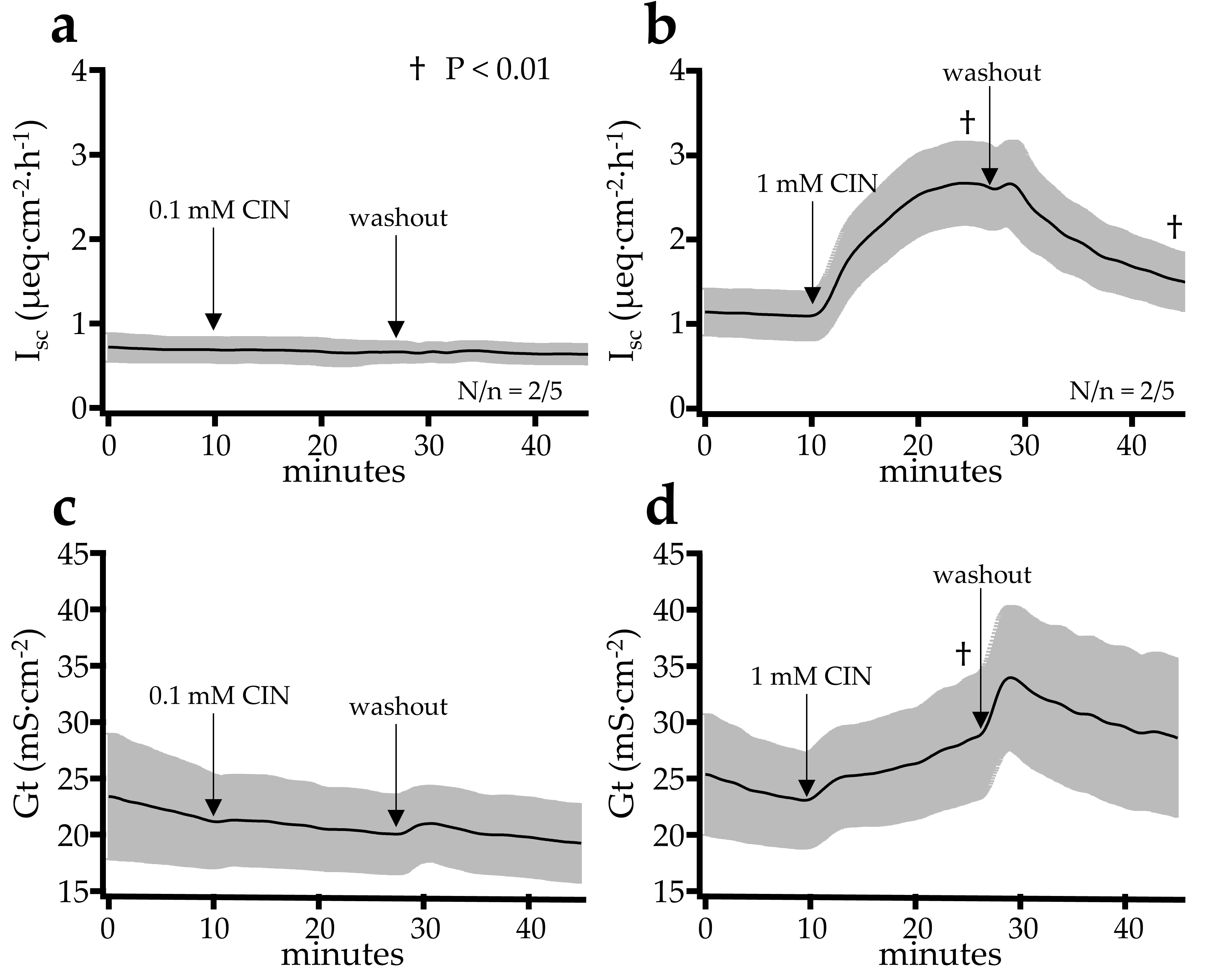

Supplement: Supplementary file 1 [file ijms-22-05198-s001.zip › Figure2.png]

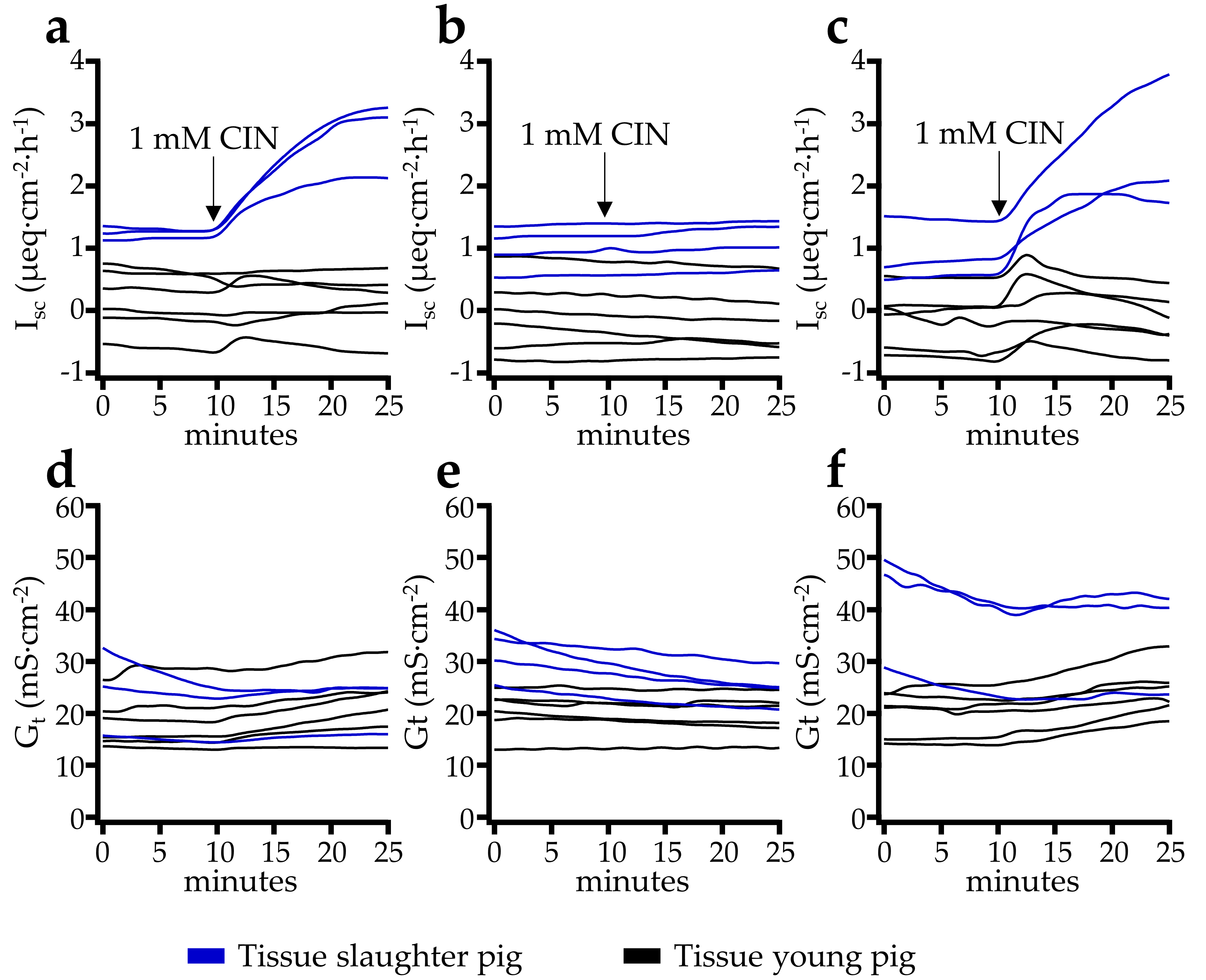

Supplement: Supplementary file 1 [file ijms-22-05198-s001.zip › Figure3.png]

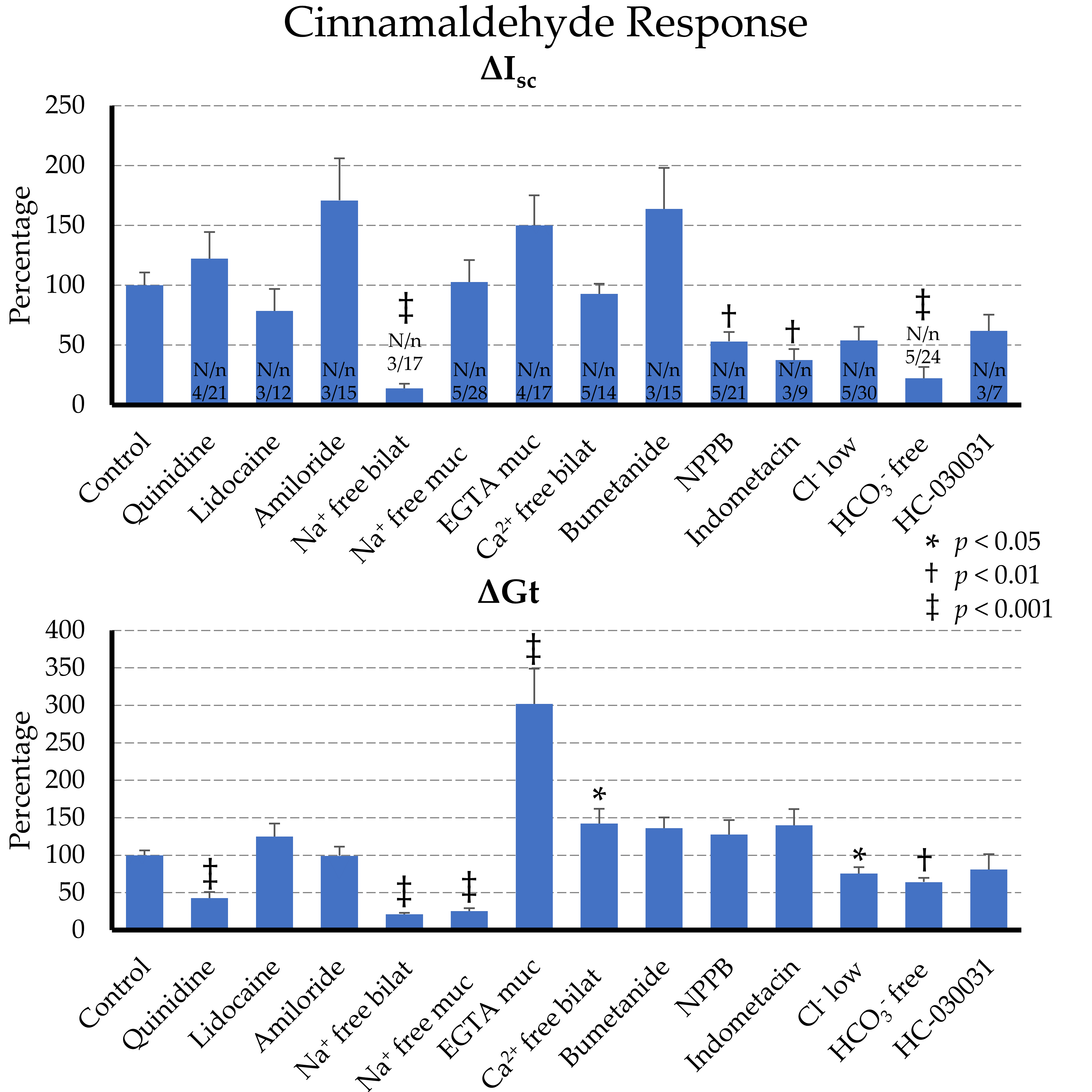

Supplement: Supplementary file 1 [file ijms-22-05198-s001.zip › Figure4.png]

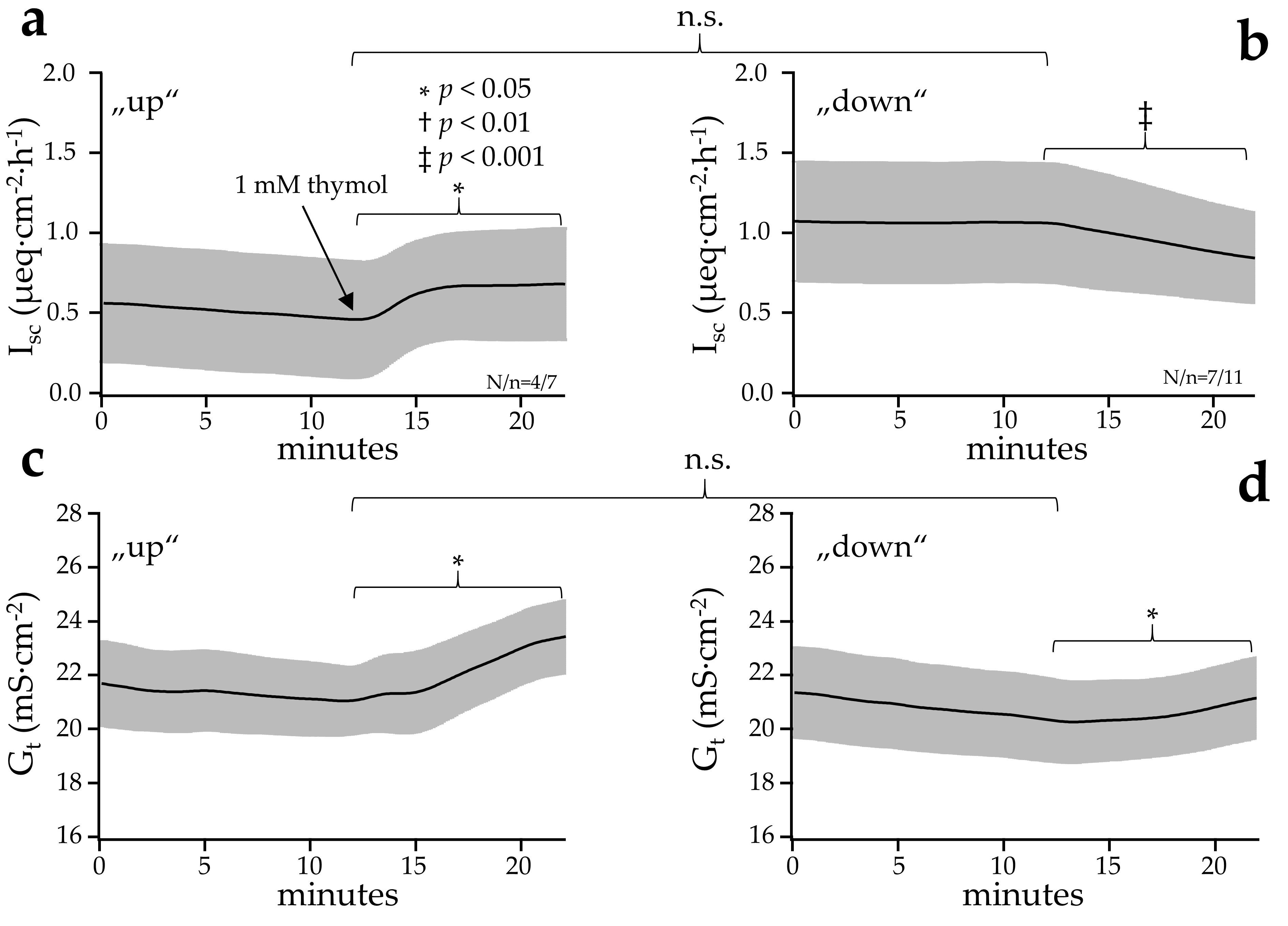

Supplement: Supplementary file 1 [file ijms-22-05198-s001.zip › Figure5.png]

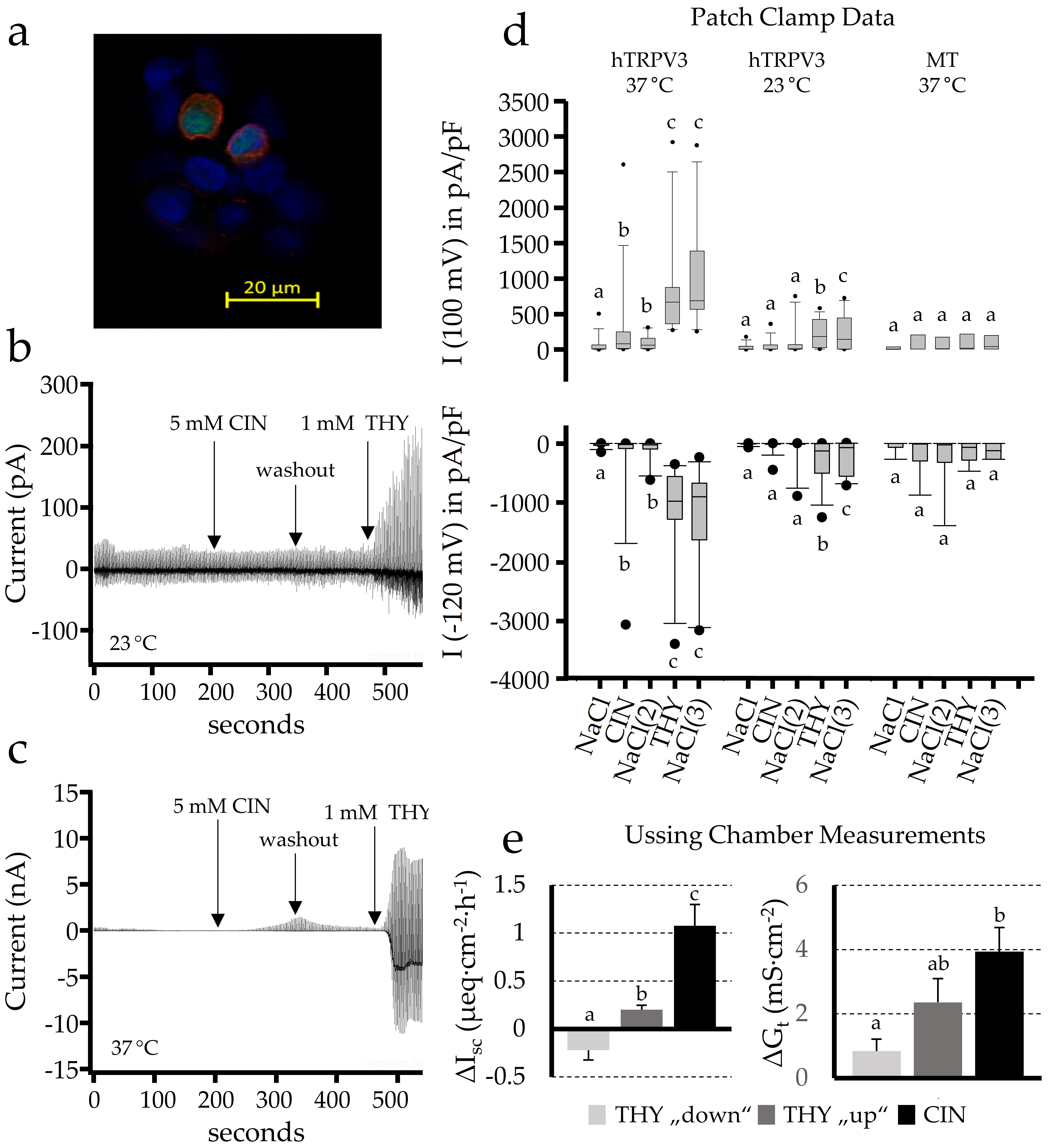

Supplement: Supplementary file 1 [file ijms-22-05198-s001.zip › Figure6.png]

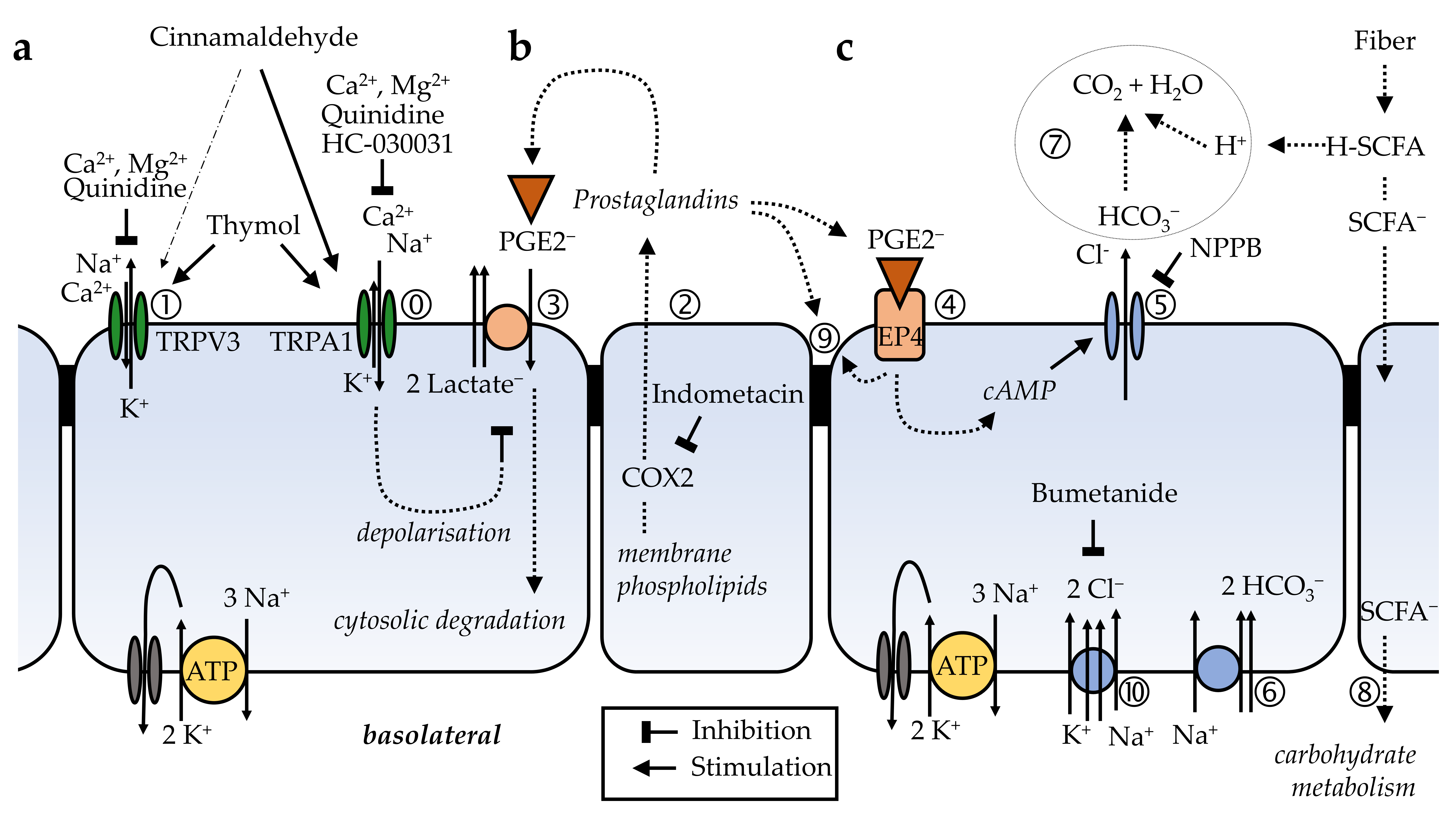

Supplement: Supplementary file 1 [file ijms-22-05198-s001.zip › Figure7.png]
